# Dissecting the Gene Expression, Localization, Membrane Topology, and Function of the Plasmodium falciparum STEVOR Protein Family

**DOI:** 10.1128/mBio.01500-19

**Published:** 2019-07-30

**Authors:** J. Stephan Wichers, Judith A. M. Scholz, Jan Strauss, Susanne Witt, Andrés Lill, Laura-Isabell Ehnold, Niklas Neupert, Benjamin Liffner, Renke Lühken, Michaela Petter, Stephan Lorenzen, Danny W. Wilson, Christian Löw, Catherine Lavazec, Iris Bruchhaus, Egbert Tannich, Tim W. Gilberger, Anna Bachmann

**Affiliations:** a Bernhard Nocht Institute for Tropical Medicine, Hamburg, Germany; b Centre for Structural Systems Biology, Hamburg, Germany; c Centre for Structural Systems Biology (CSSB), DESY, and European Molecular Biology Laboratory Hamburg, Hamburg, Germany; d Helmut-Schmidt-University, Hamburg, Germany; e Research Centre for Infectious Diseases, School of Biological Sciences, The University of Adelaide, Adelaide, South Australia, Australia; f Institute of Microbiology, University Hospital Erlangen, Erlangen, Germany; g Burnet Institute, Melbourne, Victoria, Australia; h INSERM U1016, Institut Cochin, Paris, France; i Biology Department, University of Hamburg, Hamburg, Germany; Stanford University

**Keywords:** host cell invasion, malaria, variant surface antigens

## Abstract

During its intraerythrocytic development, the malaria parasite Plasmodium falciparum exposes variant surface antigens (VSAs) on infected erythrocytes to establish and maintain an infection. One family of small VSAs is the polymorphic STEVOR proteins, which are marked for export to the host cell surface through their PEXEL signal peptide. Interestingly, some STEVORs have also been reported to localize to the parasite plasma membrane and apical organelles, pointing toward a putative function in host cell egress or invasion. Using deep RNA sequencing analysis, we characterized P. falciparum *stevor* gene expression across the intraerythrocytic development cycle, including free merozoites, in detail and used the resulting *stevor* expression profiles for hierarchical clustering. We found that most *stevor* genes show biphasic expression oscillation, with maximum expression during trophozoite stages and a second peak in late schizonts. We selected four STEVOR variants, confirmed the expected export of these proteins to the host cell membrane, and tracked them to a secondary location, either to the parasite plasma membrane or the secretory organelles of merozoites in late schizont stages. We investigated the function of a particular STEVOR that showed rhoptry localization and demonstrated its role at the parasite-host interface during host cell invasion by specific antisera and targeted gene disruption. Experimentally determined membrane topology of this STEVOR revealed a single transmembrane domain exposing the semiconserved as well as variable protein regions to the cell surface.

## Abstract (precis)

Malaria claims about half a million lives each year. Plasmodium falciparum, the causative agent of the most severe form of the disease, uses proteins that are translocated to the surface of infected erythrocytes for immune evasion. To circumvent the detection of these gene products by the immune system, the parasite evolved a complex strategy that includes gene duplications and elaborate sequence polymorphism. STEVORs are one family of these variant surface antigens and are encoded by about 40 genes. Using deep RNA sequencing of blood-stage parasites, including free merozoites, we first established *stevor* expression of the cultured isolate and compared it with published transcriptomes. We reveal a biphasic expression of most *stevor* genes and confirm this for individual STEVORs at the protein level. The membrane topology of a rhoptry-associated variant was experimentally elucidated and linked to host cell invasion, underlining the importance of this multifunctional protein family for parasite proliferation.

## INTRODUCTION

Malaria is still one of the major causes of death in developing countries. The most severe form of the disease is caused by the human malaria parasite Plasmodium falciparum. In order to ensure its survival in the human host, the parasite has evolved different immune evasion mechanisms. One of these mechanisms is the adherence of infected erythrocytes (IEs) to endothelial receptors expressed on blood vessel walls that allow the parasite to circumvent spleen passage and subsequent killing (1). This interaction between the parasite and its human host is mediated by variable surface antigens (VSAs), which are highly polymorphic and encoded by mostly subtelomere-located multicopy gene families. The exposure of different protein variants on the surface of the IE mediates antigenic variation (2). It enables the parasite to avoid recognition by the human immune system and allows the parasite to establish and maintain a long-lasting infection (1).

Although there are a number of VSA protein families, only *Pf*EMP1 (P. falciparum erythrocyte membrane protein 1) has been shown to be involved in sequestration and antigenic variation (3). However, VSAs have also been implicated in additional mechanisms of parasite survival and virulence, including rosetting (the binding of IEs to uninfected erythrocytes), platelet-mediated clumping, and the utilization of different invasion pathways to infect new host cells (2, 4, 5). It has been speculated that the VSAs RIFIN (repetitive interspersed family) (6) and STEVOR (subtelomeric variable open reading frame) (7, 8) also play a role in these processes (7).

STEVOR proteins have been shown to be involved in diverse phenomena such as changes in the rigidity of infected erythrocytes, rosette formation by binding of noninfected erythrocytes via glycophorin C, and erythrocyte invasion (9, 10). In line with the diverse biological processes STEVOR proteins are implicated in, they show multiple subcellular localizations (such as at the IE surface [10–13], Maurer’s clefts [13–16], plasma membrane [13, 17–19], and apical tip of merozoites [13, 14]) and are expressed in various stages (10, 18–22). Interestingly, expression of *stevor* genes is elevated in clinical isolates (14, 17), and absence of IE sequestration is accompanied by a lack of *stevor* expression in a splenectomized patient (23), highlighting their likely importance in parasite survival and disease severity.

STEVOR proteins share a protein structure with (i) a potential signal peptide and a PEXEL motif for protein export into the host cell at the N terminus followed by a semiconserved region (SC), (ii) a hydrophobic region, (iii) a variable protein stretch unique for each variant (V), (iv) a transmembrane region, and (v) a highly conserved C terminus. Previously, the hydrophobic region between the SC and the V region was assumed to function as an additional transmembrane domain (7), but this was challenged by recent data indicating that the SC region protrudes into the extracellular space and mediates IE binding to glycophorin C in the context of rosetting (10, 11, 13).

The reference P. falciparum strain 3D7 possesses 39 *stevor* gene copies and three *stevor*-like gene variants, which were previously shown to cluster separately in phylogenetic analyses based on differences in their amino acid composition from the putative signal sequence through the majority of the SC domain (24). One of the *stevor*-like sequences and another 9 *stevor* sequences are annotated as putative pseudogenes in the PlasmoDB database (25), but at least one of these (PF3D7_0102100) is conserved between P. falciparum isolates (26) and was shown to mediate rosetting (10), suggesting the protein is functional. Overall, 42 sequences belong to the *stevor* gene family in 3D7, with little deviation in gene numbers between different P. falciparum isolates but considerable differences in their expression (14, 17, 26). Of note, a phylogenetically closely related species, P. reichenowi, shows a considerable expansion of this family, with 66 *stevor* sequences (27). Furthermore, by comparing genomes of the *Plasmodium* subgenus *Laverania*, a strong host-specific sequence diversification could be shown for *stevor* genes, in contrast to other members of the *pir* family (28), supporting their importance in specific host-pathogen interaction.

This study provides a detailed analysis of the expression profile of all *stevor* genes using deep RNA sequencing (RNA-seq) and characterizes the localization, membrane topology, and function of selected STEVOR proteins during the intraerythrocytic developmental cycle (IDC) of the parasite.

## RESULTS

### Biphasic *stevor* expression profiles within 3D7 clones and hierarchical clustering using deep RNA-seq.

Gene expression of P. falciparum occurs in a periodic manner directly related to asexual blood-stage development. Accordingly, almost all genes have a characteristic expression window with a single peak during the IDC (29, 30). However, some particular *stevor* variants showed two expression maxima peaking in trophozoite- and merozoite-stage parasites (14).

In order to assess the transcriptional profiles of *stevor* variants across the IDC in detail, we first reviewed gene expression profiles in public databases. Our comparison of publicly available and consistently preprocessed RNA-seq gene expression data sets (31–37) revealed a high variability in *stevor* gene expression patterns between different studies, which are caused by confounding experimental factors, including experimental designs (e.g., replicates, read numbers, sequencing length, and time points of harvest), culture conditions (e.g., serum versus AlbuMAX supplementation, gas mixture, passage number, accumulated genetic variation in different cell lines, and synchronization method), and also clonal variation that has been observed for the *stevor* family (38, 39).

**FIG 1:**
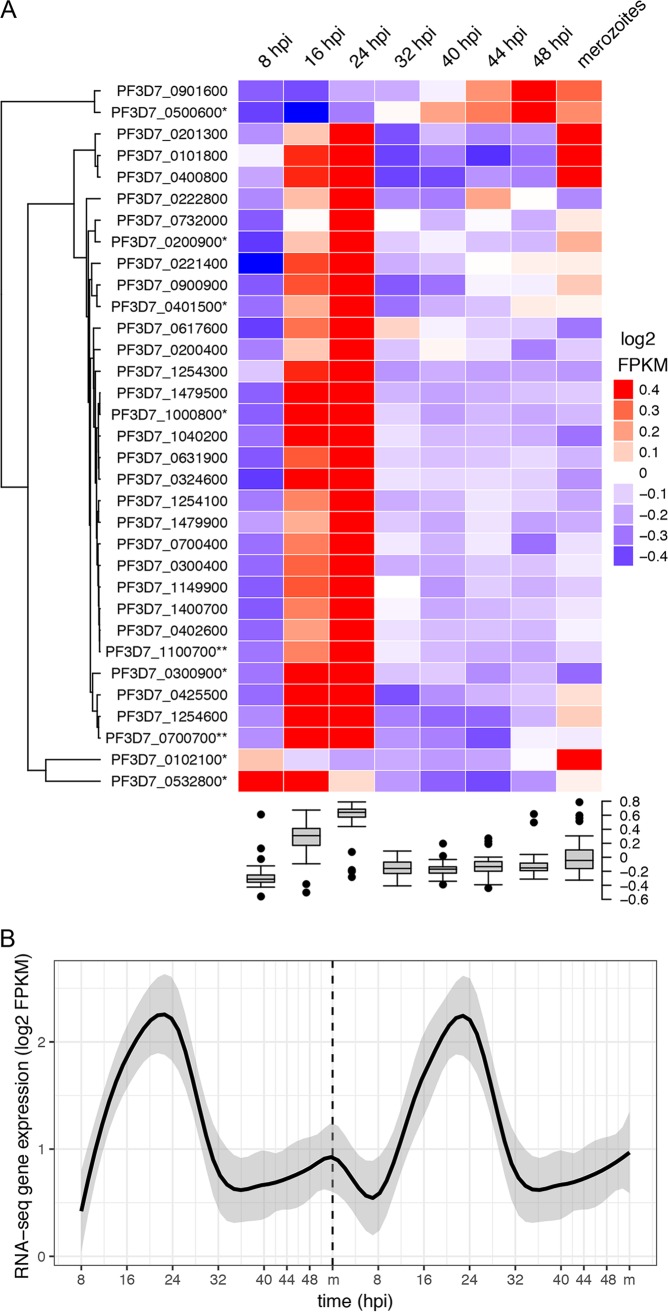
RNA-seq gene expression of *stevor* variants in Plasmodium falciparum 3D7 during the blood-stage replication cycle. (A) Hierarchical clustering heatmap of log_2_-transformed fragments per kilobase of transcript per million (FPKM) gene expression values for 33 *stevor* genes during the blood-stage cycle. The color scale ranges from blue to red, showing a range from minimal (≤−0.4) to maximal (≥0.4) log_2_ FPKM gene expression values for each *stevor* variant across the blood-stage cycle. Row labels with an asterisk denote gene models annotated as pseudogenes, and double asterisks denote gene models annotated as “*stevor*-like.” Boxplot heatmap column annotations show the variability of gene expression values for the total gene expression of all *stevor* variants at each time point. (B) Line plot showing smoothed conditional means of log_2_ FPKM *stevor* gene expression values as calculated with the LOESS smoothing method, including their 0.95 confidence intervals (gray), over two consecutive blood-stage cycles (separated by a dashed line labeled m). To visualize the biphasic expression, data were extrapolated on a second consecutive cycle.

To overcome these limitations (see Fig. S1 and Table S1 in the supplemental material) and directly link detailed gene expression profiles with functional characterization of individual *stevor* variants, we first performed an RNA-seq time course experiment. Synchronized 3D7 parasites (young ring stage [8 hpi], late ring stage/early trophozoite [16 hpi], mid-age trophozoite [24 hpi], late trophozoite [32 hpi], early schizont [40 hpi], schizont [44 hpi], late schizont [48 hpi], and purified merozoites [0 hpi]) (Fig. S2) were used to generate a reference transcription profile. To improve the discriminatory resolution of gene expression data for highly similar *stevor* gene variants (*stevor* variants show high sequence identity of 55 to 89% [average, 71.93%] at the genomic level and 15 to 85% [average, 52.53%] at the protein level across all 42 annotated *stevor* genes), we performed 100-bp paired-end RNA-seq and generated an average of 11.7 (±1.9) million paired-end reads per sample in biological triplicates (Table S2). The generated sequence data set provides a deeper coverage of the blood-stage transcriptome than previously published RNA-seq studies (Table S2), and we subsequently focused on the newly generated RNA-seq data set for the refinement of P. falciparum *stevor* gene expression. The RNA-seq data set is available in the ArrayExpress database (http://www.ebi.ac.uk/arrayexpress) under accession number E-MTAB-7731. In total, transcripts from 33 *stevor* variants were detected, indicating that 9 variants from chromosomes 1, 8, and 13 were not expressed. Subsequent gene-specific quantitative real-time PCR (qPCR) using genomic DNA (gDNA) revealed that these 9 *stevor* genes (PF3D7 gene identifiers 0114600, 0115400, 0832000, 0832400, 0832600, 0832900, 1300900, 1372500, and 1372800) are lost from the genome of the 3D7 strain used in this study (data not shown). Second, to identify similarities and differences in expression patterns for different gene variants, we performed hierarchical clustering of the individual *stevor* gene expression profiles from our RNA-seq data set that were normalized for sequencing depth and gene length using log_2_-transformed fragments per kilobase of transcript per million (FPKM) gene expression values (Fig. 1). Besides the major cluster consisting of 26 *stevor* variants with an expression maximum at the early trophozoite stage (16 hpi), we observed an additional cluster comprising three variants with two expression maxima at 24 hpi and in merozoites. Clearly separated from these main clusters were two variants (PF3D7_0901600 and PF3D7_0500600) that showed an expression maximum at the schizont stage and two variants clustering separately that showed distinct expression patterns: PF3D7_0102100 had an expression maximum in merozoites and PF3D7_0532800 had maximal expression in ring-stage parasites (Fig. 1).

10.1128/mBio.01500-19.1 FIG S1 Comparison of RNA-seq *stevor* gene expression profiles across different studies. (A) Multiple line plots of absolute gene expression values (log_2_ R/FPKM) across the blood-stage replication cycle for seven publicly available RNA-seq datasets compared to the dataset generated during the present study. All datasets were consistently processed to allow for unbiased comparison between different studies. (B) Blow-up of multiple-line plot of log_2_ FPKM *stevor* gene expression values shown in subpanel of panel A for the present study plotted over two consecutive blood-stage cycles (separated by a dashed line). To visualize the biphasic expression, data were extrapolated on a second consecutive cycle. Download FIG S1, PDF file, 0.6 MB. Copyright © 2019 Wichers et al. 2019 Wichers et al. This content is distributed under the terms of the Creative Commons Attribution 4.0 International license.

10.1128/mBio.01500-19.2 FIG S2 Time course experiment. (A) Representative images of Giemsa smears of all time points (8 hpi to 48 hpi), as well as schizonts after MACS purification and purified merozoites. (B) Quantification (number of counted iRBCs of >270 per time point) of stage distribution of all time points in the three individual replicates. (C) Quantification of stage distribution shown as means from all three replicates. Error bars show standard deviations (SD). Blue, ring-stage parasites; red, trophozoites; green, schizonts. Download FIG S2, TIF file, 0.6 MB. Copyright © 2019 Wichers et al. 2019 Wichers et al. This content is distributed under the terms of the Creative Commons Attribution 4.0 International license.

10.1128/mBio.01500-19.7 TABLE S1 Summary of metadata and experimental designs for RNA-seq datasets analyzed in this study. Download Table S1, PDF file, 0.1 MB. Copyright © 2019 Wichers et al. 2019 Wichers et al. This content is distributed under the terms of the Creative Commons Attribution 4.0 International license.

10.1128/mBio.01500-19.8 TABLE S2 Summary of RNA-seq mapping statistics for Plasmodium falciparum 3D7 RNA-seq dataset generated in this study. Download Table S2, PDF file, 0.04 MB. Copyright © 2019 Wichers et al. 2019 Wichers et al. This content is distributed under the terms of the Creative Commons Attribution 4.0 International license.

**FIG 2:**
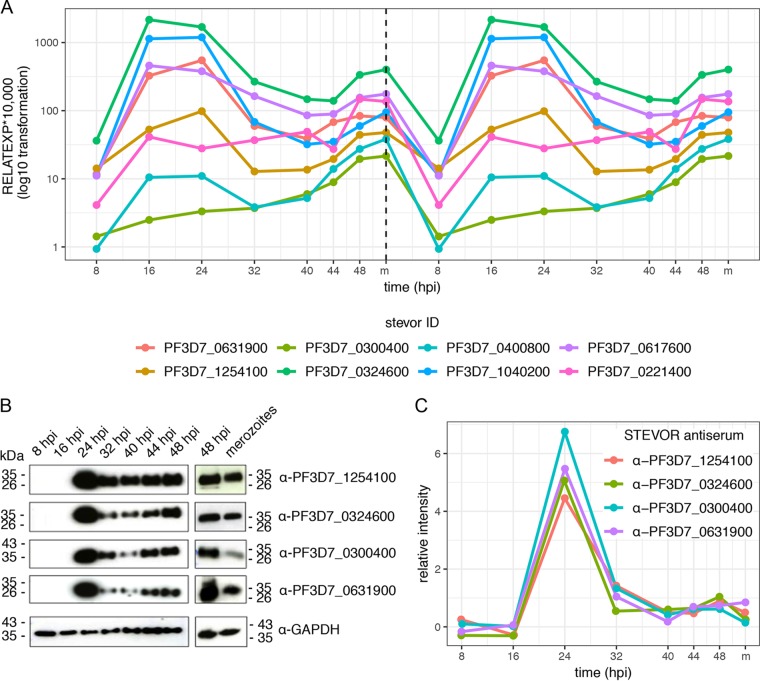
Validation of stevor expression oscillation using qPCR and Western blot analysis. (A) qPCR validation of eight *stevor* genes over a proliferation cycle. RNA expression levels were normalized to the average from two housekeeping genes (*fructose-bisphosphate aldolase* and *arginyl-tRNA synthetase)* and log_10_ transformed. To visualize the biphasic expression pattern, qPCR data were extrapolated on a second consecutive cycle. RELATEXP*10,000, relative expression * 10,000. (B) Stage-specific Western blot analysis using the STEVOR-specific anti-PF3D7_1254100, anti-PF3D7_0324600, anti-PF3D7_0300400, and anti-PF3D7_0631900 sera. An equivalent of 1 × 10^7^ cells were loaded onto each lane, and analysis with anti-GAPDH serum was used to visualize equal loading. (C) For densitometry analysis, STEVOR signal intensities were normalized to signals obtained with anti-GAPDH and calibrated against the 48-hpi time point (=1) to correct for sample variation. Note that random noise (jitter) was added to line and scatter plots to avoid overplotting.

Analysis of the variation of RNA-seq expression values of each *stevor* variant from its median expression value across the blood infection cycle indicated that 24 of the 33 *stevor* variants are actually expressed in two distinct bursts per IDC. The second expression maximum is at the schizont or merozoite stage, as indicated by an expression level above the median expression level of the respective gene (deviation from the median, ≥0.1) (Fig. S3). In contrast, the analysis of gene expression profiles of other genes showed a single stage-specific expression maximum, confirming the absence of a general biphasic expression profile across the IDC (Fig. S4). The robustness of our RNA-seq analysis was validated by qPCR with primers targeting 8 different *stevor* variants, confirming the timing of these variants through the cell cycle (Fig. 2A). This common second expression maximum for most of the *stevor* genes at the late schizont/merozoite stage has not been identified in other published studies omitting gene expression analysis of the free merozoite stage (Fig. S1).

10.1128/mBio.01500-19.3 FIG S3 Variation of *stevor* gene expression in Plasmodium falciparum 3D7 during the blood-stage replication cycle. Heatmap showing values for the deviations of log_2_ FPKM RNA-seq expression values of each *stevor* variant from its median expression value across the blood-stage infection cycle. The order of *stevor* variants is according to hierarchical clustering in Fig. 1. The color scale ranges from blue to red, indicating minimal (−2) and maximal (6) values for deviations from the respective median log_2_ FPKM expression values. Row labels with an asterisk denote gene models annotated as “*stevor*-like,” and double asterisks denote gene models annotated as pseudogenes. Download FIG S3, PDF file, 2.5 MB. Copyright © 2019 Wichers et al. 2019 Wichers et al. This content is distributed under the terms of the Creative Commons Attribution 4.0 International license.

10.1128/mBio.01500-19.4 FIG S4 Gene expression over the IDC for genes with a nonbiphasic expression profile. Multiple-line plots of RNA-seq gene expression profiles showing log_2_-transformed FPKM expression values of glyceraldehyde-3-phosphate dehydrogenase (PF3D7_1462800; GAPDH), aldolase (PF3D7_1444800), early transcribed membrane protein 2 (PF3D7_0202500; ETRAMP2), membrane-associated histidine-rich protein 1 (PF3D7_1370300; MAHRP1), plasmoredoxin (PF3D7_0303600; PLRX), FP2a (PF3D7_1115700), merozoite surface protein 1 (PF3D7_0930300; MSP1), and apical membrane antigen 1 (PF3D7_1133400; AMA1). To visualize the biphasic expression, data were extrapolated on a second consecutive cycle. Genes were split into subgroups according to their stage-specific expression to assist visualization. Download FIG S4, PDF file, 2.3 MB. Copyright © 2019 Wichers et al. 2019 Wichers et al. This content is distributed under the terms of the Creative Commons Attribution 4.0 International license.

### Oscillation of STEVOR expression and differential localization.

Biphasic gene expression of STEVORs could enable a single variant to perform different functions during the IDC. We confirmed biphasic gene expression on the protein level using specific antisera for four selected STEVORs with similar gene expression profiles: PF3D7_0631900, PF3D7_1254100, PF3D7_0324600, and PF3D7_0300400 (Fig. 2B). Sequence specificity of the antisera was confirmed using transgenic parasite lines expressing the selected STEVOR as either green fluorescent protein (GFP)- or hemagglutinin (HA)-tagged variants (Fig. S5A to C). For semiquantitative Western blot analysis, parasite lysates were harvested in parallel to RNA samples, and signal intensities were quantified densitometrically and normalized to a glyceraldehyde-3-phosphate dehydrogenase (GAPDH) loading control (Fig. 2C). The antisera detected a single protein of approximately 35 kDa in Western blot analysis corresponding to the calculated molecular weight of 35 to 37 kDa (Fig. 2B). These results mirror the RNA expression pattern but with a time delay in protein abundance caused by translational processes and show a biphasic protein expression pattern with the highest protein levels in early trophozoites (24 h postinfection), a subsequent drop in protein levels, and then a gradual increase of the signal intensity until the late schizont stage. A significant amount of STEVOR protein was also found in purified merozoites but to a lesser extent than that in schizonts.

10.1128/mBio.01500-19.5 FIG S5 Validation of rabbit anti-STEVOR sera. (A) Western blot of ama1-STEVOR-GFP cell lines and of 3D7 and episomally GFP-tagged cell lines (EPI). Shown is detection of ama1-STEVOR (PF3D7_0631900, PF3D7_1254100, PF3D7_0324600, and PF3D7_0300400)-GFP using rabbit antisera directed against the indicated STEVOR variant and anti-GFP antibody (Roche). Anti-PF3D7_0300400 antiserum detects additional proteins in Western blotting but clearly recognized the specific STEVOR-GFP fusion in the transgenic parasite line. Anti-aldolase antibodies have been used as a loading control. (B) Western blot detection of crt_PF3D7_0324600-GFP/-3xHA cell line. Shown is Western blot detection of 3D7 and crt_PF3D7_0324600-GFP/-3xHA cell lines using rabbit antisera directed against PF3D7_0324600, anti-GFP antibody (Roche), and anti-HA antibody (Roche). (C) Western blot detection of crt_PF3D7_0631900-GFP/-3xHA cell line. Shown is Western blot detection of 3D7 and crt_PF3D7_0631900-GFP/-3xHA cell lines using rabbit antisera directed against PF3D7_0631900, anti-GFP antibody (Roche), and anti-HA antibody (Roche). (D) Localization of crt_PF3D7_0324600-GFP/-3xHA cell line. Shown is microscopy of unfixed cells and IFA of crt_PF3D7_0324600-GFP/-3xHA using rabbit antisera directed against PF3D7_0324600, anti-GFP antibody (Roche), anti-HA antibody (Roche), and anti-SBP1 antibody. (E) Localization of crt_PF3D7_0631900-GFP/-3xHA cell line. Shown is microscopy of unfixed cells and IFA of crt_PF3D7_0631900-GFP/-3xHA using rabbit antisera directed against PF3D7_0631900, mouse anti-GFP antibody (Roche), rat anti-HA antibody (Roche), and rabbit anti-SBP1 antibody. (F) Localization of crt_PF3D7_1254100-GFP and PF3D7 0300400-GFP cell lines. Shown is microscopy of unfixed crt_PF3D7_1254100-GFP and crt_PF3D7_0300400-GFP parasites. Anti-aldolase antibodies were used as a loading control for Western blotting. Nuclei were stained with DAPI or Hoechst as indicated. Scale bars, 1 μm. Download FIG S5, TIF file, 2.9 MB. Copyright © 2019 Wichers et al. 2019 Wichers et al. This content is distributed under the terms of the Creative Commons Attribution 4.0 International license.

**FIG 3:**
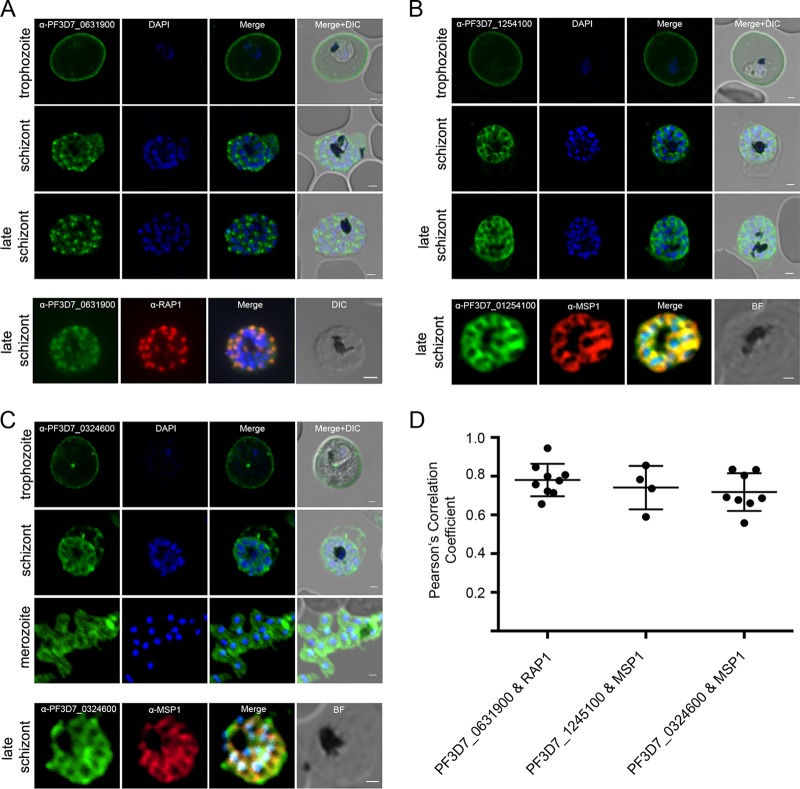
Localization of selected STEVOR variants. Individual STEVORs were localized either in trophozoites, schizonts, or merozoites using anti-PF3D7_0631900 (A), anti-PF3D7_1254100 (B), and anti-PF3D7_0324600 (C) sera. Anti-MSP1 or anti-RAP1 antibodies were used for colocalization experiments. DIC, differential interference contrast. (D) Colocalization was quantified using the JACOP plugin (97) for ImageJ, and Pearson’s correlation coefficients were calculated for anti-PF3D7_0631900/anti-RAP1 of 0.780 (±0.084), anti-PF3D7_1245100/anti-MSP1 of 0.741 (±0.112), and anti-PF3D7_0324600/anti-MSP1 of 0.718 (±0.098). Nuclei were stained with DAPI. Scale bars, 1 μm. BF, bright field.

We then analyzed the subcellular localization of these 4 individual STEVOR proteins using the variant-specific anti-STEVOR sera across the time course of the IDC. As previously reported for PF3D7_1040200, PF3D7_0617600, and PF3D7_1254100 (10, 11, 13), all selected STEVOR variants are localized at the erythrocyte membrane in trophozoite stages (Fig. 3A to C and Fig. S6A). However, in late schizonts and merozoites a differential STEVOR localization was apparent. PF3D7_1254100 and PF3D7_0324600 localized at the merozoite plasma membrane in late-stage schizonts and free merozoites, colocalizing with the merozoite membrane marker merozoite surface protein 1 (MSP1) (Fig. 3B to D). In late schizonts, PF3D7_0631900 localized predominantly at the apical tip of merozoites and colocalized with the rhoptry bulb marker rhoptry-associated protein 1 (RAP1) (Fig. 3A and D). The localization of PF3D7_0300400 resulted in an ambiguous and weak staining pattern in schizonts that may be due to lower gene expression of this particular STEVOR (Fig. S6A). The differential localization of PF3D7_1254100 at the merozoite plasma membrane and PF3D7_0631900 at the apical tip in late-stage parasites was confirmed in transgenic parasites overexpressing the individual STEVOR variants as GFP-tagged chimeras under the late-stage-specific apical membrane antigen 1 (*ama1*) promoter (Fig. S6B). In contrast to the antibody-based or HA-tagged localization, STEVOR-GFP fusions localized to the Maurer’s clefts in trophozoites. This suggests that the GFP tag hampers the correct export of STEVORs to the erythrocyte plasma membrane (Fig. S5D to F).

10.1128/mBio.01500-19.6 FIG S6 (A) Localization of PF3D7_0300400. Shown is IFA detection of 3D7 parasites (trophozoite and schizont) using rabbit anti-PF3D7_0300400 serum. Nuclei were stained with DAPI. Scale bars, 1 μm. (B) Localization of ama1-PF3D7_0631900-GFP and ama1-PF3D7_1254100-GFP. Shown is microcopy of unfixed ama1-PF3D7_0631900-GFP and ama1-PF3D7_1254100-GFP late-stage schizonts. Nuclei were stained with Hoechst. Scale bars, 1 μm. (C) Mouse and rat antisera directed against PF3D7_0631900 are able to reduce parasite proliferation. Effect on parasite proliferation of mouse and rat antisera directed against PF3D7_0631900 was assessed in a growth inhibition assay using different antiserum concentrations. In parallel, pooled sera from naïve mice (grey dashed lines) and the corresponding rat preimmune serum (black dashed line) were tested as indicated. Data were tested for normal distribution via Shapiro-Wilk test, and statistical significances were determined with an independent *t* test (*, *P* < 0.05). Experiments were performed in triplicates using biologically independent samples. Error bars show standard deviations. Confocal imaging used mouse antiserum against PF3D7_0631900 (green) in trophozoites and late schizonts. Detection of PF3D7_0631900 used IFA and Western blotting for both the 3D7 and crt-PF3D7_0631900–GFP (EPI) samples using rat antiserum directed against PF3D7_0631900. Rat anti-PF3D7_0631900 detects additional proteins in Western blotting but clearly recognized the specific STEVOR-GFP fusion in the transgenic parasite line. Anti-aldolase antibodies have been used as a loading control. Scale bars, 1 μm. (D and E) Growth curves of PF3D7_0631900-TGD. Shown are growth curves of three individual experiments, starting with tightly synchronized ∼0.1% parasitemia cultures of 3D7 (blue) and PF3D7_0631900-TGD (red) parasites, grown at 37°C for 4 days, and parasitemia was measured every 24 h by flow cytometry. Growth in percent relative to that of 3D7 wild-type control is shown in panel E. Experiments were performed in triplicates using biologically independent samples. Error bars show standard errors of the means (SEM). Download FIG S6, TIF file, 1.4 MB. Copyright © 2019 Wichers et al. 2019 Wichers et al. This content is distributed under the terms of the Creative Commons Attribution 4.0 International license.

Overall, STEVOR proteins localize to different subcellular locations within the infected erythrocyte, and this differs depending on the life cycle stage and STEVOR variant. The selected STEVORs are present at the erythrocyte membrane in trophozoite stages and in schizonts either at the parasite plasma membrane (PF3D7_1254100 and PF3D7_0324600) or the rhoptries (PF3D7_0631900) of the erythrocyte-invading merozoite.

### Antisera generated against STEVOR variant PF3D7_0631900 inhibit parasite growth by interfering with the invasion process.

**FIG 4:**
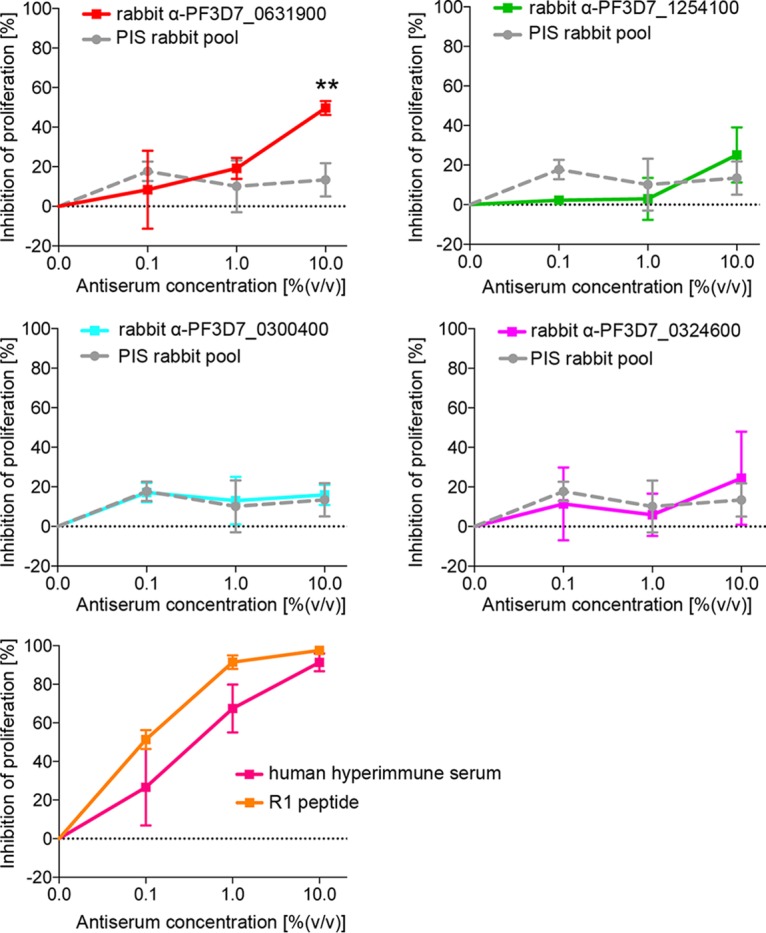
Proliferation inhibition. Effect on parasite proliferation in the presence of different concentrations of anti-PF3D7_0631900 (red), anti-PF3D7_1254100 (green), anti-PF3D7_0300400 (turquoise), or anti-PF3D7_0324600 (magenta) sera. In parallel, pooled sera from naive animals (gray dashed lines) were tested as indicated. A human hyperimmune serum (23) with a serum titer of antibodies against P. falciparum of >1:5,000 (pink) as well as the R1 peptide (40) blocking AMA1 (orange) were used as positive controls, while preimmune sera (PIS) were used as negative controls. Data were tested for normal distribution via the Shapiro-Wilk test, and statistical significance was determined with an independent *t* test (**, *P* < 0.01). Experiments were performed in triplicates using biologically independent samples. Error bars show standard deviations.

The localization of PF3D7_1254100 and PF3D7_0324600 at the plasma membrane of the merozoite and of PF3D7_0631900 at the rhoptries points toward a putative role in either egress or invasion. First, the growth-inhibitory potentials of antisera directed against all four selected STEVORs were assayed (Fig. 4). While antisera directed against PF3D7_1254100, PF3D7_0324600, and PF3D7_0300400 showed no significant inhibition, parasite growth was significantly reduced using the antiserum directed against the rhoptry-localized PF3D7_0631900 (49.79 ± 3.58; means ± standard deviations [SD]) (Fig. 4). This specific growth inhibition for antisera directed against PF3D7_0631900 can be confirmed using antisera raised in both mice (66.18 ± 8.82) and rats (61.17 ± 3.78) (Fig. S6C). A human hyperimmune serum assumed to recognize a broad STEVOR repertoire with known inhibitory capacity (23) (91.31 ± 4.62) and the R1 inhibitory peptide targeting AMA1 function (40) (97.60 ± 1.21) were included as positive controls for inhibition of invasion, while preimmune sera were used as negative controls (13.45 ± 8.45).

**FIG 5:**
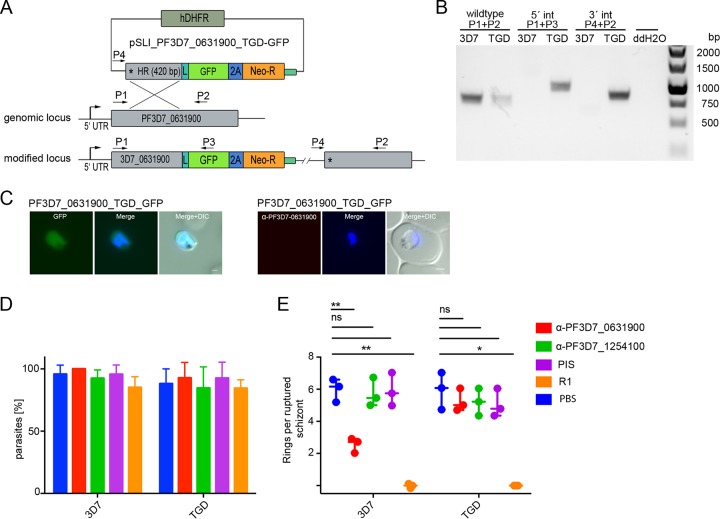
Invasion inhibition by targeting individual STEVOR proteins. (A) Schematic representation of the PF3D7_0631900 knockout strategy using the pSLI-TGD vector system (88). Dark green, human dihydrofolate dehydrogenase (hDHFR); gray, homology region (HR); green, green fluorescence protein (GFP) tag; blue, T2A skip peptide (105, 106); orange, neomycin resistance cassette. Stars indicate stop codons, and arrows depict primers (P1 to P4) used for the integration check PCR. UTR, untranslated region. (B) PCR analysis of the rendered genomic locus using primers as shown in the schema using gDNA of 3D7 wild-type and PF3D7_0631900-TGD parasites. ddH_2_O, distilled water. int, integration. (C) Localization of the truncated PF3D7_0631900-GFP and IFA using anti-PF3D7_0631900 antibodies. Nuclei were stained with Hoechst (unfixed cells) and DAPI (fixed cells). Scale bars, 1 μm. (D and E) Effect on egress (percentage of ruptured schizonts) (D) and invasion (number of rings per ruptured schizont) (E) of anti-PF3D7_0631900 and anti-PF3D7_1254100. Preimmune serum pool, PBS, and the R1 peptide were applied as controls. Statistical significance (ns, not significant; *, *P* < 0.05; **, *P* < 0.01) were determined by performing a paired *t* test. Experiments were performed in triplicates on different days using biologically independent samples. Error bars show standard deviations. TGD, PF3D7_0631900-TGD.

To distinguish between a function in schizont egress or erythrocyte invasion of the PF3D7_0631900 STEVOR protein, we aimed to analyze the ratio between ruptured schizonts and newly invaded ring-stage parasites in the presence and absence of the inhibitory antiserum anti-PF3D7_0631900. As an additional specificity control, we generated a functional PF3D7_0631900 null mutant (TGD) (Fig. 5A and B) by truncating and GFP tagging the endogenous STEVOR gene, resulting in a protein devoid of a transmembrane domain. As expected, the truncated version of PF3D7_0631900 devoid of a transmembrane domain exclusively localized within the cytosol of the infected erythrocyte (Fig. 5C). We used this cell line side by side with the wild-type 3D7 parasite line in an invasion inhibition assay (Fig. 5D and E). Tightly synchronized parasites were incubated for 12 h with anti-PF3D7-0631900 serum, and the ratio between ruptured schizonts and newly invaded parasites was analyzed. As negative controls, anti-PF3D7_1254100 serum (showing no proliferation inhibitory phenotype) or preimmune sera and phosphate-buffered saline (PBS) were used. As shown in Fig. 5D and E, incubation with anti-PF3D7_0631900 serum, but not anti-PF3D7_1254100 serum or the preimmune serum, reduces the number of newly invaded ring-stage parasites per ruptured schizont from 5.99% ± 0.72% IEs to 2.55% ± 0.46% IEs, corresponding to a 55.50% ± 4.69% reduction in invasion (Fig. 5E). No significant changes in the ruptured schizont-stage parasites were observed (Fig. 5D), strongly arguing for an invasion-inhibitory effect of this antiserum. By comparison, the blocking of AMA1 function using the R1 peptide reduces the number of newly invaded ring-stage parasites per ruptured schizont by 96.77 ± 1.25%. Importantly, the inhibitory effect of anti-PF3D7_0631900 serum is lost with the genetic disruption of this STEVOR variant (Fig. 5E).

### STEVOR membrane topology.

**FIG 6:**
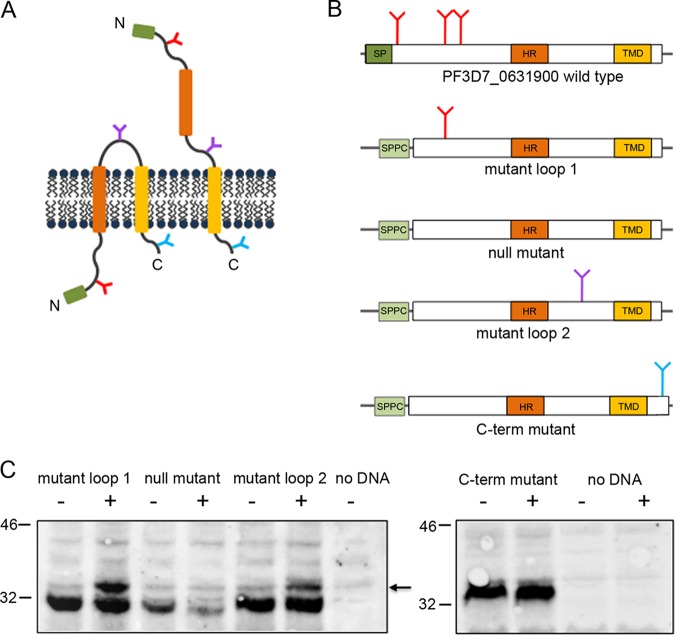
Topology studies of PF3D7_0631900 variants. (A) Proposed topology models of STEVOR proteins: two-transmembrane domain topology with the C and N termini localized on the same membrane side (N_in_-C_in_, left) and one-transmembrane topology with both domains on opposite sides (N_out_-C_in_, right). The signal peptide (SP) is indicated by a green box, the hydrophobic region (HR) (putative transmembrane domain) is indicated by a brown box, and the proposed transmembrane domain (TMD) is indicated by an orange box. Native and engineered N-glycosylation acceptor sites are indicated by red, purple, and blue Ys. (B) Schematic presentation of the PF3D7_0631900 wild type and PF3D7_0631900 glycosylation variants containing efficiently glycosylated N-glycosylation sites in loop 1 or loop 2 or at the C terminus. Loop mutants in a nonglycosylated state correspond to a molecular weight of 31 kDa or 33 kDa in the case of the C-terminal glycosylation mutant. The artificial signal peptide (SPPC) is colored in light green. All other color coding is according to that used for panel A. (C) Topology mapping of the constructs described for panel B by expressing them in a cell-free *in vitro* rabbit reticulocyte lysate in the absence (−) and presence (+) of canine rough microsomes (CRM). The expected size for glycosylated species is indicated with a black arrow. As a negative control, the assay was performed without adding any DNA in the absence or presence of CRM.

To further probe the function of the rhoptry-associated STEVOR PF3D7_0631900 in erythrocyte invasion, we investigated its membrane topology. Despite the importance of membrane topology for the function of the STEVOR protein family, a model for the topology of STEVORs has yet to be validated and accepted. While all tested transmembrane prediction tools (Phobius [41], TMHMM [42, 43], and TOPCONS [44]) suggest two transmembrane domains, which supports a topology with both termini being located on the intracellular side of the membrane, a one-transmembrane topology model with an extracellular N terminus has also been proposed (13, 45) (Fig. 6A). To test these predictions experimentally for the PF3D7_0631900 variant, we employed a microsome-supplemented *in vitro* system (46). In this system, glycosylation occurs in the lumen of canine pancreas rough microsomes (CRMs) so that only protein regions that translocate to the lumen are specifically glycosylated. We engineered expression plasmids in which the native STEVOR N terminus was replaced by an artificial signal peptide (47) to ensure transport to and translocation into the microsomal membrane reporter system (48). Proteins for all constructs were expressed in a cell-free rabbit reticulocyte lysate in the absence or presence of CRMs. We engineered STEVOR variants with a single N-glycosylation acceptor site located in regions between the artificial signal peptide and the central hydrophobic region, a putative transmembrane domain (mutant loop 1) between the central hydrophobic region and the C-terminal transmembrane domain (mutant loop 2), or at the C-terminal end (C-term mutant) (Fig. 6B; also see Materials and Methods). To increase the specificity of specific glycosylation events, two of the three native glycosylation sites in the N-terminal domain were mutated in these constructs to aid in detection of specific glycosylation events. As shown in Fig. 6C, bands of increased molecular weight consistent with the 2-kDa increase in protein size, indicative of glycosylation, can be observed only in the cases of the loop 1 and loop 2 mutants in the presence of CRM. No such species can be observed in the case of the C-terminal mutant, indicating that this region was external to the CRM lumen. As the glycosylation efficiency is within the range routinely obtained in our *in vitro* system, we conclude that the higher-molecular-weight species represent glycosylated versions of the STEVOR variants. This suggests that the N terminus all the way up to the transmembrane domain must be translocated into the lumen of the microsomes for glycosylation to occur efficiently. The lack of glycosylation in case of the C-terminal mutant strongly suggests that the N and C termini of PF3D7_0631900 are located at opposite sides of the microsomal membrane. This supports the finding that the hydrophobic region toward the N terminus does not act as a transmembrane domain and that a one-transmembrane topology (N_out_-C_in_) of PF3D7_0631900 within various membranes is likely. Hence, the entire N-terminal region, including the hydrophobic region, could be involved in host-pathogen interaction.

## DISCUSSION

The present study provides an in-depth analysis of the expression of all members of the *stevor* multicopy gene family as well as the localization, membrane topology, and function of selected STEVOR proteins across the P. falciparum IDC. Our new high-resolution RNA-seq data set extends previously published RNA-seq data sets and represents a useful resource for transcriptional analysis of the entire blood-stage transcriptome of P. falciparum. Accordingly, detailed transcriptional profiles were inferred from our new RNA-seq data set, showing that most genes possess only a single, well-defined expression window during the IDC (see Fig. S4 in the supplemental material) (29, 30). In contrast, our RNA-seq data set provides strong evidence for a biphasic expression profile of most *stevor* genes, which can be found in the main cluster based on expression profiles. This biphasic expression was previously described for only four *stevor* variants, with a second peak of expression in merozoites detected by microarray and confirmed for PF3D7_0631900 and PF3D7_1254100 via qPCR.

It is interesting that 9 telomere-localized *stevor* genes out of 39 annotated in the 3D7 reference strain are lost in the 3D7 line used in this study. These chromosomal telomeric deletions are a previously documented phenomenon in clonal parasite lines (50–52) that can result in a spectrum of deletion genotypes (26). However, the detected chromosomal deletions in our 3D7 line as well as the engineered knockout of PF3D7_0631900 shows no significant decrease in proliferation under normal cell culture conditions (Fig. S6D). These data underline the functional redundancy and plasticity of *stevor* genes and reveal a different phenotype reported from a saturation-based mutagenesis screen (53) published recently for the P. falciparum isolate NF54.

We then were interested in investigating the subcellular localizations of STEVORs in different stages and their specific function. We analyzed four selected STEVOR proteins with highly similar transcriptional profiles using, to our knowledge for the first time, well-characterized STEVOR variant-specific antisera in conjunction with corresponding transgenic parasite lines. In agreement with numerous previous studies, we observed a localization of all variants at the erythrocyte membrane in trophozoite stages, underlining their putative function in cell-cell interactions (9–11, 13, 26, 54). Similar to our results, GFP-tagged STEVOR versions were previously shown to be retained at the Maurer’s clefts, indicating that the interaction of the STEVOR C terminus with the erythrocyte cytoskeleton (55) is hampered by GFP tagging and precludes transport to the IE membrane (15). Cell lines overexpressing a STEVOR variant with a smaller tag reflected the localization observed by antibody staining, which confirms IE membrane staining of STEVORs during the trophozoite stages (26). Interestingly, trafficking of STEVORs to different compartments of the merozoite seems not to be influenced by the GFP tag. In schizont stages, divergent subcellular loci of individual STEVORs were apparent with either plasma membrane or rhoptry localization. Merozoite plasma membrane localization was previously reported using a polyclonal mouse anti-PF3D7_1254100 serum that targets a conserved STEVOR region and thus does not discriminate between individual variants (13, 18, 19). Here, we provide additional evidence that PF3D7_1254100 is indeed localized at the merozoite plasma membrane using a different antiserum as well as transgenic parasites. Furthermore, using the same approach we identified the *stevor* variant PF3D7_0324600 as the variant localizing at the merozoite plasma membrane. Interestingly, this variant was previously predicted to be an adhesin (56). We also establish PF3D7_0631900 as a novel member of apically localized STEVORs, as indicated previously for PF3D7_1040200 (14) and PF3D7_1300900 (13), and provide evidence that this STEVOR variant is a rhoptry-associated protein. It is unclear how these exported proteins can overwrite the PEXEL export signature and end up in distinct localizations, such as in the plasma membrane or the rhoptries. One can speculate that in the newly formed progeny merozoites in late-schizont-stage parasites the protein export machinery is not yet established, which would result in blocked export to the host cell and lead to the alternative localization of the STEVORs.

The potential to inhibit merozoite invasion into new host cells using antisera directed against four STEVOR variants was analyzed. Using our PF3D7_0631900-specific antisera in conjunction with our transgenic PF3D7_0631900 functional knockout cell line, we provide the first evidence for the role of a particular STEVOR at the parasite-host interface during erythrocyte invasion, expanding the initial work by Niang and colleagues, who used sera that recognized at least two variants and inhibited parasite growth to a lesser extent (10, 11). Consistent with other studies, an effect on erythrocyte invasion could only be obtained with antisera detecting an apically localized STEVOR variant, whereas antibodies targeting variants with merozoite plasma membrane localization showed no influence on parasite growth (10, 19). Of note, other studies showed similar observations for members of another multicopy gene family, named *surf*, where antibodies directed against a particular SURFIN (SURFIN_4.2_), which is located at the merozoite plasma membrane and at the rhoptries, were able to partially inhibit invasion (57–59). Alternatively, a steric inhibition of, e.g., other invasion-related receptors by the STEVOR-bound antibodies could also explain our results. Although rhoptry proteins have mainly been implicated in events central to invasion, we cannot rule out that the PF3D7_0631900 STEVOR variant and maybe other VSAs also are important downstream of parasite entry into the new host cell already observed for other rhoptry-located proteins (60). Blocking of this variant could also impair PV (parasitophorous vacuole) development, leading to the observed growth effect in 3D7 wild-type cells. This would also be in line with the absence of a growth effect upon knockout of PF3D7_0631900 but could also be explained by intra- and interfamily redundancy within these multicopy gene families. This redundancy could also be exploited by the parasite during the selection process of the PF3D7_0631900 knockout cell line, leading to its functional compensation. In this light it is interesting that a general knockdown of either the *stevor*, *rif*, or *pfmc-2tm* (P. falciparum *Maurer’s clefts 2 transmembrane*) gene via promoter titration also did not reveal a proliferation phenotype *in vitro* but showed an influence of the expression of the other two gene families (61). Interestingly, PF3D7_0631900 is conserved (>99% sequence identity) in two recently published field isolates (P. falciparum GA01 and KE01) (62).

How PF3D7_0631900 and other STEVOR proteins contribute to erythrocyte invasion or function early in the newly invaded erythrocyte remains unknown. A first step to understanding the precise molecular function of these proteins is the elucidation of their membrane topology. Here, we took advantage of a microsome-supplemented *in vitro* expression system recently used to confirm the single-transmembrane topology for RIFIN proteins (63). Our results indicate that PF3D7_0631900 possesses a one-transmembrane topology, supporting the findings of a previous study that used subcellular fractionation and trypsinization assays to investigate STEVOR topology (13). It is conceivable that the N-terminal domain of PF3D7_0631900 and all other STEVOR proteins, given their canonical domain architecture, forms the extracellular portion of the protein responsible for protein-protein interactions and is involved in direct or indirect interactions with erythrocyte surface structures. Two recent studies deliver additional data confirming the topology we deduced from our experiments. Zhou et al. showed that patient-derived antibodies indeed predominantly target the predicted surface-exposed domains of STEVOR proteins (64). Andersson et al. also employed N-linked glycosylation of sites within two other STEVOR variants (PF3D7_0617600 and PF3D7_1040200), revealing the same topology (65).

In conclusion, this study highlights that many STEVOR proteins are transcriptionally active at multiple stages in the IDC, suggesting that they play important roles in different stages of parasite development. We show that different STEVOR variants localize to distinct subcellular localizations in the merozoite, where they may contribute to different cellular processes. Identifying that a merozoite-expressed STEVOR had a classical one-transmembrane topology supports the suggestion that the N terminus of these antigens could be exposed to the extracellular environment. Importantly, the growth-inhibitory action of antibodies directed against the N terminus of the STEVOR variant PF3D7_0631900 demonstrates that merozoite-expressed STEVORs can be targeted by vaccine-induced antibodies.

## MATERIALS AND METHODS

### Cultivation and sampling of parasites for RNA-seq and immunoblot analyses.

The P. falciparum clone 3D7 (66) was cultivated at a hematocrit of 5% in human O^+^ erythrocytes according to standard procedures (67). To obtain tightly synchronized parasites, 3D7 cultures were treated twice with 5% sorbitol (68) 6 h apart, and the resulting schizonts were separated using the magnetically activated cell sorting (MACS) column system. Uninfected O^+^ erythrocytes were added to allow reinvasion during subsequent cultivation. After 8 h, the culture was synchronized with 5% sorbitol, which allows only ring-stage parasites to survive (4 hpi ± 4 hpi). Samples for RNA-seq and immunoblot analyses were taken after the next reinvasion at 8, 16, 24, 32, 40, 44, and 48 hpi to avoid the influence of sorbitol treatment on RNA data. Additionally, merozoites were purified mechanically through filtration from E64-treated schizonts after 52 h (69). For RNA analyses, erythrocyte pellets were rapidly lysed in 10× to 20× volumes of prewarmed TRIzol (Life Technologies) and stored at −70°C until RNA purification. The time course expression experiment was repeated at an interval of 4 weeks to obtain three independently cultivated biological samples for RNA-seq.

### RNA extraction, RNA-seq library preparation, and sequencing.

RNA purification and DNase treatment of the samples was performed as described previously (70). Human globin mRNA was depleted from the samples using the GLOBINclear kit (Life Technologies) according to the manufacturers’ instructions. RNA quantity was assessed using the Qubit RNA HA assay kit and a Qubit 3.0 fluorometer (ThermoFischer Scientific), and quality was assessed using the Agilent RNA 6000 Pico kit with the Bioanalyzer 2100 (Agilent). To cope with the high AT content of P. falciparum, the cDNA library was amplified using the KAPA HiFi polymerase in the presence of tetramethylammonium chloride (TMAC) (71). HiSeq 4000 100-bp paired-end sequencing was performed with 30% lane control by BGI Genomics Co. (Hong Kong) (72). On average, 23.5 (±4) million clean reads were obtained for individual samples.

The LightCycler 480 (software version 1.5; Roche) was used for quantitative real-time PCR analysis to confirm the absence of gDNA in the RNA-seq samples. Fifty ng of total RNA was mixed with QuantiTect SYBR green PCR reagent (Qiagen) and 0.3 μM sense and antisense primers targeting *fructose-bisphosphate aldolase* (PF3D7_1444800) (see Table S3 in the supplemental material) (73) in a final volume of 10 μl. Reaction mixtures were incubated at 95°C for 15 min and then subjected to 40 cycles of 95°C for 15 s and 60°C for 1 min and a subsequent melting step (60 to 95°C). The specificity of the primer pair was confirmed after each qPCR run by dissociation curve analysis. Threshold calculation was done using the fit points analysis method provided by the LightCycler 480 software, release 1.5.1.62 SP3. If necessary, DNase treatment was repeated until the qPCR run was clean.

10.1128/mBio.01500-19.9 TABLE S3 Oligonucleotides used in this study. Download Table S3, PDF file, 0.1 MB. Copyright © 2019 Wichers et al. 2019 Wichers et al. This content is distributed under the terms of the Creative Commons Attribution 4.0 International license.

### RNA-seq read mapping and data analysis.

After successful quality checks of RNA-seq reads with FastQC v0.11.5 (http://www.bioinformatics.babraham.ac.uk/projects/fastqc/) (74), reads were aligned to the P. falciparum 3D7 genome v3.0, release 34, available from the PlasmoDB genome database (25), using the RNA-seq aligner STAR v2.5.3a (75). Prior to aligning reads, STAR was used to generate a genome index using the downloaded genome sequence fasta file (PlasmoDB-34_Pfalciparum3D7_Genome.fasta) and GFF3 annotation file (PlasmoDB-34_Pfalciparum3D7.gff). Mapped reads in Sequence Alignment/Map (SAM) format were then summarized using the featureCounts (76) function of the Rsubread v1.24.2 R package (77). To establish the optimal filtering parameters used for the STAR alignment and featureCounts read summarization, a range of maximum allowed mismatches (STAR option, –outFilterMismatchNmax 0|1|2|5|10) and a range of minimum fragment lengths (featureCounts option, minFragLength = 0|50|75|80|85|90|100|200|300) were tested for the STAR alignments for counting mapped reads for genomic features. Informed by experimental evidence that nine *stevor* variants (PF3D7 gene identifiers 0114600, 0115400, 0832000, 0832400, 0832600, 0832900, 1300900, 1372500, and 1372800) are absent from the investigated P. falciparum 3D7 laboratory strain, the maximum allowed mismatches was set to one (–outFilterMismatchNmax 1) during STAR alignments, and a minimum fragment length of 85 bp (minFragLength = 85) was chosen to count mapped reads for genomic features using featureCounts when processing data for all downstream sequence analysis. Results from initial quality checks with FastQC and STAR alignments were summarized in single report files using MultiQC v1.2 (78).

The R package edgeR (79) was used to compute FPKM using its rpkm function. The gene lengths of transcripts required for FPKM normalization were extracted from the PlasmoDB-34_Pfalciparum3D7_AnnotatedTranscripts.fasta file by indexing it using SAMtools (80) faidx and extracting the first two columns containing sequence name and length. The gene expression profiles were visualized with heatmaps using the ComplexHeatmap R package (81) using a one minus Pearson's correlation distance metric and the average linking method to cluster *stevor* genes. Line plots were generated with ggplot2 (82). Specifically, line plots of smoothed conditional means of log_2_ FPKM *stevor* gene expression values and their 0.95 confidence intervals were calculated with a default locally estimated scatterplot smoothing (LOESS) smoother using a span of 0.3 to control the amount of smoothing.

A statistical analysis of summarized read counts per genomic feature obtained from featureCounts (76) was performed with the R package ALDEx2 (83, 84) to carry out an analysis of variance (ANOVA)-like differential gene expression analysis. Prior to the analysis, the data were separated into replicated sample pairs based on sampling condition (i.e., time point) to allow for multiple pairwise comparisons of statistical effect sizes between successive time points. The centered log-ratio (clr)-transformed posterior distribution then was generated by 128 Monte Carlo replicates drawn from a Dirichlet distribution using the interquartile log-ratio (iqlr) approach for each pairwise comparison as implemented in the aldex.clr function of ALDEx2 (83, 84). Differential abundance tests were then conducted using the glm test for one-way ANOVA, returning Benjamini-Hochberg corrected *P* values, and three significance levels (*, *P* < 0.05; **, *P* < 0.01; ***, *P* < 0.001) are reported for individual *stevor* genes that are significantly regulated across the blood-stage cycle. Finally, *post hoc* testing was performed by estimating effect sizes with the aldex.effect function for successive pairwise effect size comparisons between consecutive time points, considering absolute effect sizes of >2 and overlap of less than 0.01 as highly significant changes in gene expression between time points (Table S4A and B).

10.1128/mBio.01500-19.10 TABLE S4 Results of ANOVA-like differential gene expression testing for multiple pairwise comparisons between successive time points using ALDEx2. Download Table S4, PDF file, 0.1 MB. Copyright © 2019 Wichers et al. 2019 Wichers et al. This content is distributed under the terms of the Creative Commons Attribution 4.0 International license.

Publicly available RNA-seq data sets (Table S1) were downloaded from public read archives, and raw fastq files were consistently processed as described above for comparative analysis.

### qPCR.

qPCR was performed for 8 *stevor* variants to validate RNA-seq results using 50 ng cDNA and qPCR mix in a final volume of 10 μl. Reaction mixtures were incubated at 95°C for 15 min and then subjected to 40 cycles of 95°C for 15 s and 60°C for 1 min with a subsequent melting step (60 to 95°C). RNA from the second biological sample of the time course experiment was reverse transcribed using SuperScript III with random hexamers (Life Technologies). An amplification efficiency of over 1.9 was determined by dilution of a single gDNA over 5 to 6 logs of concentration for each primer pair used (Table S3). *stevor* expression was corrected for amplification efficiency, normalized against the average expression of the two housekeeping genes *fructose-bisphosphate aldolase* (PF3D7_1444800) and *arginyl-tRNA synthetase* (PF3D7_1218600), and calibrated against gDNA.

qPCR was performed using specific primers for 41 of the 42 *stevor* variants (except PF3D7_0300900) (Table S3) in order to confirm their presence in or absence from the genome of 3D7. qPCR reactions were performed using 2.5 ng gDNA and qPCR mix in a final volume of 10 μl. Cycling was done as described above.

### Nucleic acids and constructs.

All oligonucleotides used for plasmid construction are listed in Table S3.

For recombinant expression and subsequent immunization of the 4 *stevor* variants (PF3D7_0324600, amino acids [aa] 53 to 262; PF3D7_1254100, aa 24 to 248; PF3D7_0300400, aa 33 to 251; PF3D7_0631900, aa 53 to 263), the N-terminal parts of the genes were amplified using 3D7 genomic DNA and cloned into the HindIII/BamHI restriction site of the protein expression plasmid pJC45 (85).

For generation of transgenic parasites overexpressing the *stevor* genes PF3D7_0324600, PF3D7_1254100, and PF3D7_0300400, full-length coding regions were synthesized (GenScript, USA). For PF3D7_0631900, pARL-STEVOR^full^, a plasmid containing the variant PF3D7_0631900, was kindly provided by Jude Przyborski (15). Twenty-four of the 27 missing base pairs (without the TAA stop codon) at the C terminus were added by amplification using an extended reverse primer. For all four variants KpnI/AvrII restriction sites were added by PCR, and the PCR products were digested and inserted into the KpnI/AvrII sites of the pARL-1a-GFP or pARL-1a-3xHA plasmid. Expression of the fusion protein is driven by either the *ama1* (86) promoter to mimic expression in schizonts or the *crt* (87) promoter to mimic expression in trophozoites.

For targeted gene disruption of PF3D7_0631900, a 420-bp homology region was amplified from 3D7 gDNA and cloned into pSLI-TGD (88) using the NotI/MluI restriction site.

For *in vitro* translation assays, the gene coding for PF3D7_0631900 was amplified by PCR from the previously described pARL-ama1-PF3D7_0631900-GFP construct using 5′- and 3′-specific oligonucleotides encoding either an artificial signal peptide (MAKLLLLLLLLLLPSAQA [47]) and the mature N terminus after PEXEL cleavage or the wild-type C terminus, as well as containing suitable restriction sites for cloning into an optimized pRSET expression plasmid that featured a mutated Kozak sequence for enhanced translation and an A_30_ tail as described in the Promega technical note (45). Due to the exchange of the native N terminus of PF3D7_0631900 for the artificial signal peptide, the resulting PF3D7_0631900 variant contains two native potential N-glycosylation acceptor site (Asn-Xaa-Thr/Ser) motifs located in the region upstream of the first predicted transmembrane domain (loop 1). For further topology studies, both loop 1 N-glycosylation acceptor sites were deleted by mutagenesis and an individual additional site (Asn-Xaa-Thr) was engineered in the region between the two predicted transmembrane domains (loop 2). A variant containing a unique (Asn-Xaa-Thr) site at the C terminus was also engineered. Steric hindrance of the glycosylation process at the C-terminal end due to proximity to the transmembrane domain was avoided by adding a 19-amino-acid C-terminal extension containing the glycosylation acceptor site. Site-specific mutagenesis was performed according to the method of Kunkel (89, 90). All mutants were confirmed by sequencing of plasmid DNA.

### Transfection of P. falciparum.

For transfection, late-schizont-stage parasites were transfected with 50 μg of plasmid DNA using Amaxa Nucleofector 2b (Lonza, Switzerland) as previously described (91). Transfectants were selected using 4 nM WR99210 (Jacobus Pharmaceuticals). Gene deletion of PF3D7_0631900 was achieved using the pSLI-TGD vector system (88). In order to select for mutant cell lines using the SLI-TGD system, G418 at a final concentration of 400 μg/ml (ThermoFisher, USA) was added to a 5% parasitemia culture. The selection process and testing for integration were performed as previously described (88).

### Antisera and immunoblots.

Recombinant proteins were produced in Escherichia coli as previously described (92) (Table S3). Antisera were raised in mice and rabbits (Pineda Antikörper-Service, Germany) (93). In addition, synthesis of recombinant protein and immunization of rats with PF3D7_0631900 was performed by Eurogentec (Belgium) using the 28-day speedy immunization protocol.

Immunoblots were performed using saponin-lysed, infected erythrocytes. Parasite proteins were separated on a 12% SDS-PAGE gel using standard procedures (13) and transferred to a nitrocellulose membrane (Amersham Protran; 0.45-μm-pore-size nitrocellulose membrane; GE Healthcare) using a Trans-Blot device (Bio-Rad) according to the manufacturer’s instructions. Anti-STEVOR sera, rabbit anti‐glyceraldehyde‐3‐phosphate dehydrogenase (anti-GAPDH) (a kind gift of C. Daubenberger), and rabbit anti-aldolase (94) were diluted 1:2,000, mouse anti-GFP (Roche) antibody was diluted 1:500 or 1:1,000, and rat anti-HA (Roche) antibody was diluted 1:1,000 or 1:2,000. No signal was obtained for the STEVOR preimmune sera diluted 1:2,000.

The chemiluminescent signal of the horseradish peroxidase-coupled secondary antibodies (Dianova) was visualized either on an Amersham Hyperfilm ECL (GE Healthcare) or using a Chemi Doc XRS imaging system (Bio-Rad) and processed with Image Lab 5.2 software (Bio-Rad). To perform loading controls and ensure equal loading of parasite material, anti-GAPDH or anti-aldolase antibodies were used. The corresponding immunoblots were incubated two times in stripping buffer (0.2 M glycine, 50 mM dithiothreitol, 0.05% Tween 20) at 55°C for 1 h and washed 3 times with Tris-buffered saline for 10 min. To quantify immunoblot signal densities, three different blot films were digitalized and analyzed with ImageJ 1.50i (95). Data incorporating the area of the STEVOR-specific protein band as well as the intensity of the signal were normalized to the loading control GAPDH and calibrated against the 48-hpi time point.

### Cell-free *in vitro* expression and microsomal membranes.

Purified plasmids encoding truncated PF3D7_0631900 glycosylation mutants were used as templates for *in vitro* expression experiments. The experiments were performed using the TNT quick coupled transcription/translation kit (Promega, Madison, WI) in the presence or absence of canine pancreas microsomes according to the manufacturer’s protocols.

Because N-linked glycosylation occurs only in the lumen of the microsomes, the localization of the introduced acceptor sites can be determined by assaying their glycosylation status. Addition of a single N-linked oligosaccharide to the nascent chain leads to an increase in molecular mass of about 2 kDa that is detectable by SDS-PAGE.

Proteins were analyzed by SDS-PAGE and blotted on nitrocellulose membranes as described above and probed with rabbit anti-PF3D7_0631900 antiserum.

### Imaging and immunofluorescence analysis (IFA).

All fluorescence images were observed and captured using either a Zeiss Axioskop 2plus microscope with a Hamamatsu Digital camera (model C4742-95) or an Olympus FV1000 confocal microscope.

Microscopy of unfixed IEs was performed as previously described (96). Briefly, parasites were incubated in RPMI 1640 culture medium with Hoechst-33342 (Invitrogen) for 15 min at 37°C prior to imaging. Seven μl of IEs was added on a glass slide and covered with a cover slip.

IFAs were performed as described previously (13). Briefly, IEs were smeared on slides and air dried. Cells were fixed in 100% ice-cold methanol or ice-cold 60:40 methanol:acetone for 3 min at −20°C. Afterwards, cells were blocked with 5% milk powder for 30 min. Primary antibodies next were diluted in PBS–3% milk powder and incubated for 2 h, followed by three washing steps in PBS. Secondary antibodies were applied for 2 h in PBS–3% milk powder containing 1 μg/ml Hoechst-33342 (Invitrogen), followed by 3 washes with PBS. One drop of mounting medium (Mowiol 4-88 [Calbiochem]) was added and the slide sealed with a coverslip for imaging.

Colocalization analysis was conducted using Pearson’s coefficients of “Just Another Colocalization Plugin” (JACoP) (97) for ImageJ in Fiji (98). Thresholds were set to the same levels of fluorescence present in the original image. The intensity correlation of the red (*y* axis) and green (*z* axis) signals was calculated, and Pearson’s coefficients were calculated for *n* (*n* = 9 for anti-PF3D7_0631900, *n* = 4 for anti-PF3D7_1245100, and *n* = 8 for anti-PF3D7_0324600) selected regions of interest, and the calculated means with respective standard deviations are shown. Antisera were used at the following dilutions: 1:100 mouse anti-PF3D7_0631900, 1:200 rabbit anti-PF3D7_0631900, 1:200 rabbit anti-PF3D7_0300400, 1:200 rabbit anti-PF3D7_1254100, 1:300 rabbit anti-0324600, 1:200 rat anti-PF3D7_0631900, 1:100 or 1:200 mouse anti-GFP (Roche), 1:500 rat anti-HA (Roche), 1:500 mouse anti-MSP1 (99), 1:1,000 mouse RAP1 (100), and 1:500 rabbit a-SBP1 (94). Nuclei were stained with Hoechst-33342 (Invitrogen) or 4′,6-diamidino-2-phenylindole (DAPI) (Roche).

Images were processed using either Photoshop (Adobe Photohop CS3 v10 Extended) or Imaris (Bitplane Imaris x64 v7.8) for display purposes only.

Compound 2, a protein kinase G inhibitor, was stored in glycerol at −20°C and used at a final concentration of 1 μM in order to arrest parasite egress before exoneme/microneme secretion for microscopy of late schizonts, as previously described (101).

### Parasite growth assays by flow cytometry.

For growth assays, parasite cultures were adjusted to equal parasitemia (0.1 to 0.3%). Parasitemia was measured every 24 h for 4 days with a flow cytometry assay adapted from previously published assays (88, 102, 103).

Briefly, for growth assay 1, P. falciparum cultures were resuspended and 20 μl was transferred to an Eppendorf tube. Eighty μl RPMI containing SYBRGreen (Sigma-Aldrich) and dihydroethidium (DHE) (ThermoFischer) then was added to obtain final concentrations of 0.25× and 5 μg/ml, respectively. Samples were incubated for 20 min (protected from UV light) at room temperature, and parasitemia was determined using a NovoCyte 1000 (ACEA Biosciences Inc.). For every sample, 100,000 events were recorded, and parasitemia was determined with NovoExpress software.

Briefly, for growth assays 2 and 3, parasite cultures were resuspended and 20-μl samples were transferred to an Eppendorf tube. Eighty μl RPMI containing Hoechst and dihydroethidium (DHE) then was added to obtain final concentrations of 5 μg/ml and 4.5 μg/ml, respectively. Samples were incubated for 20 min (protected from UV light) at room temperature, and parasitemia was determined using an LSRII flow cytometer by counting 100,000 events using the FACSDiva software (BD Biosciences).

### Proliferation inhibition assay.

One hundred μl of tightly synchronized 3D7 schizont culture with 0.5% parasitemia and a hematocrit of 5% was incubated with 0.1%, 1%, and 10%, vol/vol, antiserum or 0.01, 0.1, and 1 mg/ml R1 invasion inhibitory peptide (40) for 44 h under P. falciparum cell culture conditions. In addition to the R1 peptide, a human hyperimmune serum with a serum titer of antibodies against P. falciparum (titer of >1:5,000) (23) was used as an additional positive control. After incubation with the antiserum or peptide, parasitemia of each well was determined by blinded counting of at least 1,000 cells of three randomly selected sight fields of a Giemsa-stained smear. The whole assay was performed three times, antisera were tested in duplicates, and the analysis was performed blinded. Data were tested for normal distribution via Shapiro-Wilk test, and statistical significances were determined with an independent *t* test.

### Invasion/egress assay.

First, Percoll purification (104) of 3D7 or PF3D7_0631900-TGD synchronized late schizont culture was performed, followed by an incubation period of 3 h at 37°C on a shaker, which allowed the parasites to reinvade new erythrocytes. Sorbitol synchronization (68) then was performed in order to obtain a tightly synchronized parasite culture with a time window of a maximum of 3 h. These parasites next were grown until they reached late schizont stage again at ∼44 hpi. This tightly synchronized 3D7 or PF3D7_0631900_TGD schizont culture was then set to 1% parasitemia at a hematocrit of 5%, and 150 μl for each condition was incubated with 10 vol% PBS (1/10 the final volume, negative control), 10 vol% anti-PF3D7_0631900 serum, 10 vol% anti-PF3D7_1254100 serum, 10 vol% preimmune sera, or 10 vol% R1 invasion inhibitory peptide (positive control) (40). After 12 h of incubation, the numbers of ring-stage and schizont parasites were determined by counting at least 1,000 cells per condition in randomly selected sight fields on Giemsa smears. Ruptured schizonts and number of rings per ruptured schizont were calculated with the following equations: ruptured schizonts = [(no. of schizonts at 0 h/no. of RBCs at 0 h) – (no. of schizonts at 12 h/no. of RBCs at 12 h)]/(no. of schizonts at 0 h/no. of RBCs at 0 h) and no. of rings per ruptured schizont = [(no. of rings at 12 h/no. of RBCs at 12 h) – (no. of rings at 0 h/no. of RBCs at 0 h)]/[(no. of schizonts at 0 h/no. of RBCs at 0 h) – (no. of schizonts at 12 h/no. of RBCs at 12 h)].

The assay was repeated three times, and the analysis was performed blinded. Statistical significance (*P* < 0.05) was determined by performing a paired *t* test in Prism6 (GraphPad).

### Data availability.

RNA-seq data are available in the ArrayExpress database (http://www.ebi.ac.uk/arrayexpress) under accession number E-MTAB-7731.
