# Supplementary figures and images for: Dissecting the Gene Expression, Localization, Membrane Topology, and Function of the Plasmodium falciparum STEVOR Protein Family
Source: mBio. 2019 Jul 30;10(4):e01500-19. doi: 10.1128/mBio.01500-19 (PMC6667621; doi:10.1128/mBio.01500-19)

Figure S1

A

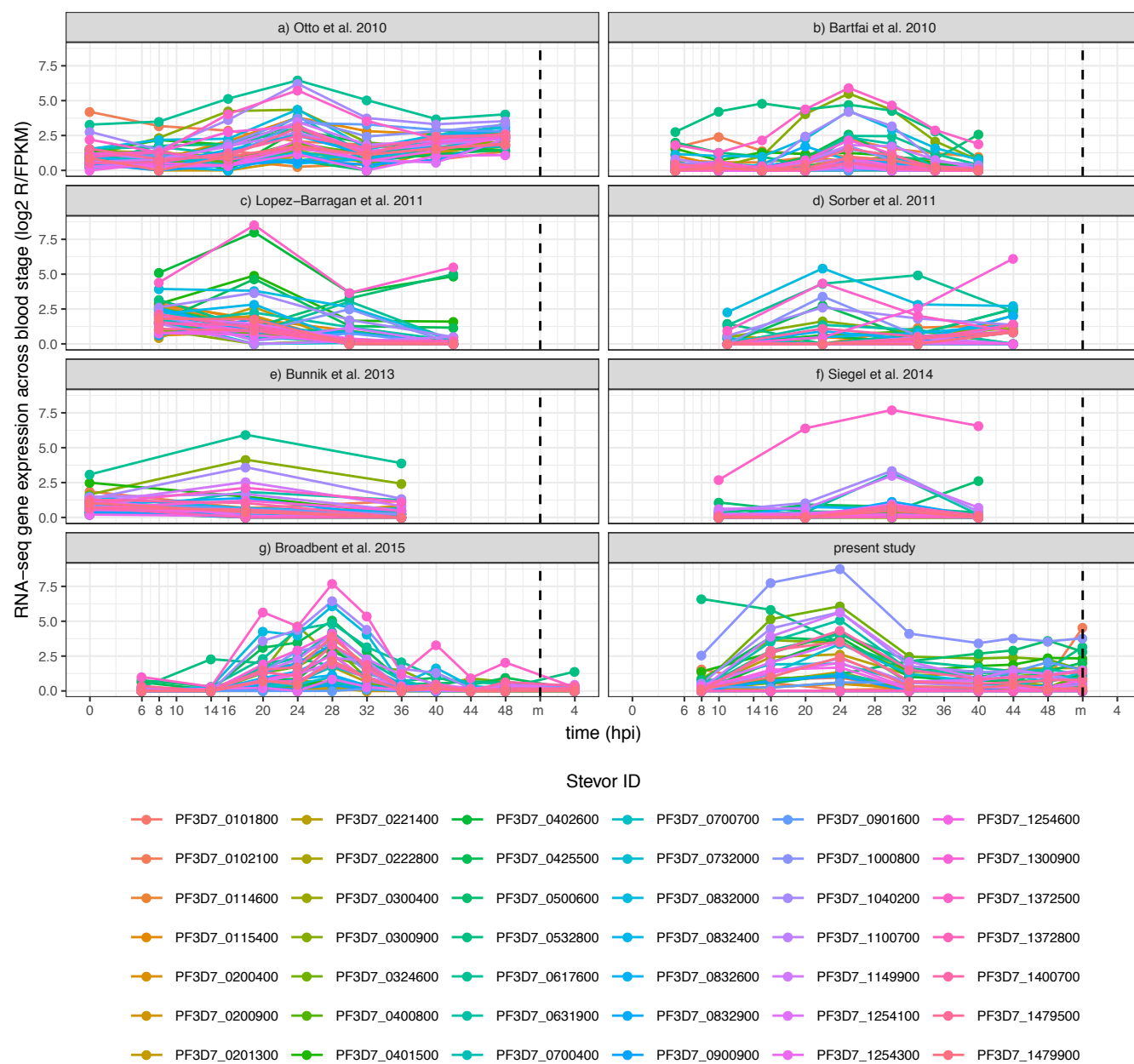

B

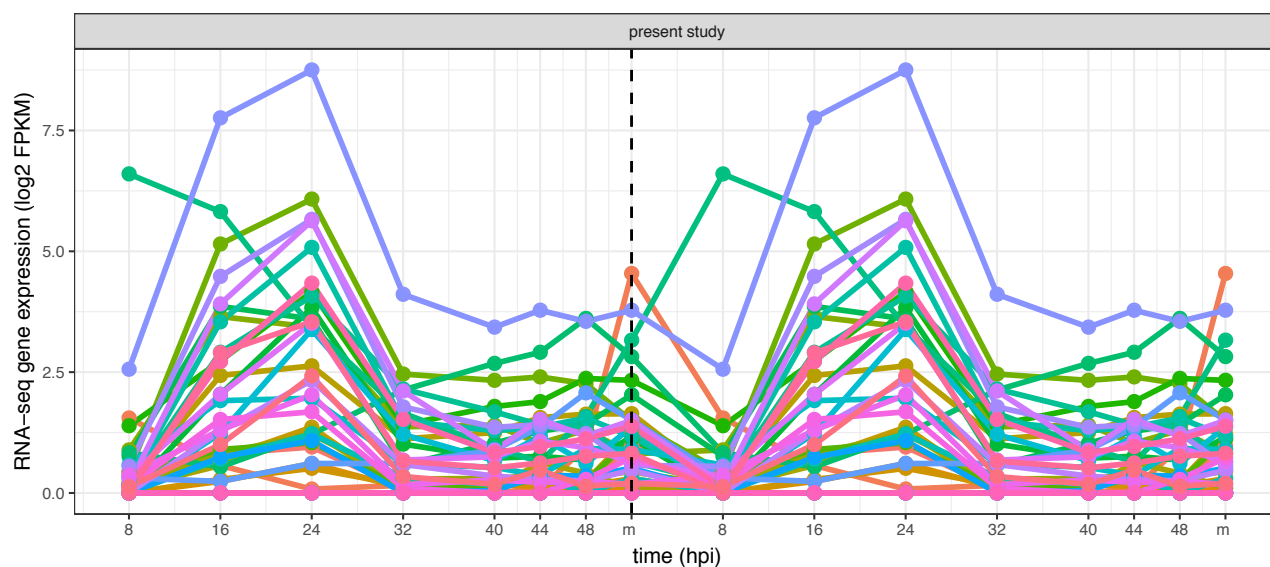

Supplement: FIG S1 [file mBio.01500-19-sf001.pdf]

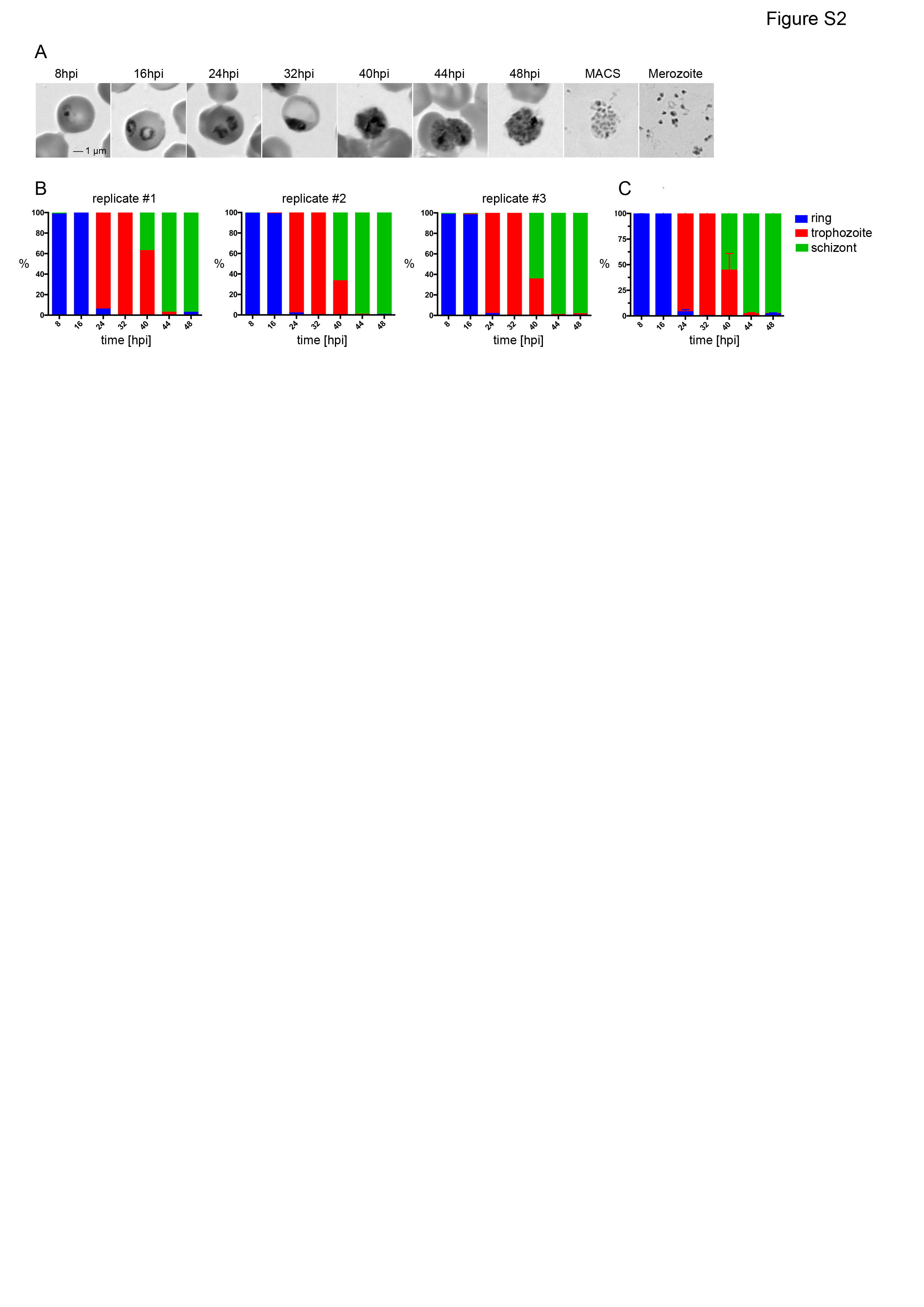

Supplement: FIG S2 [file mBio.01500-19-sf002.tif]

Figure S4

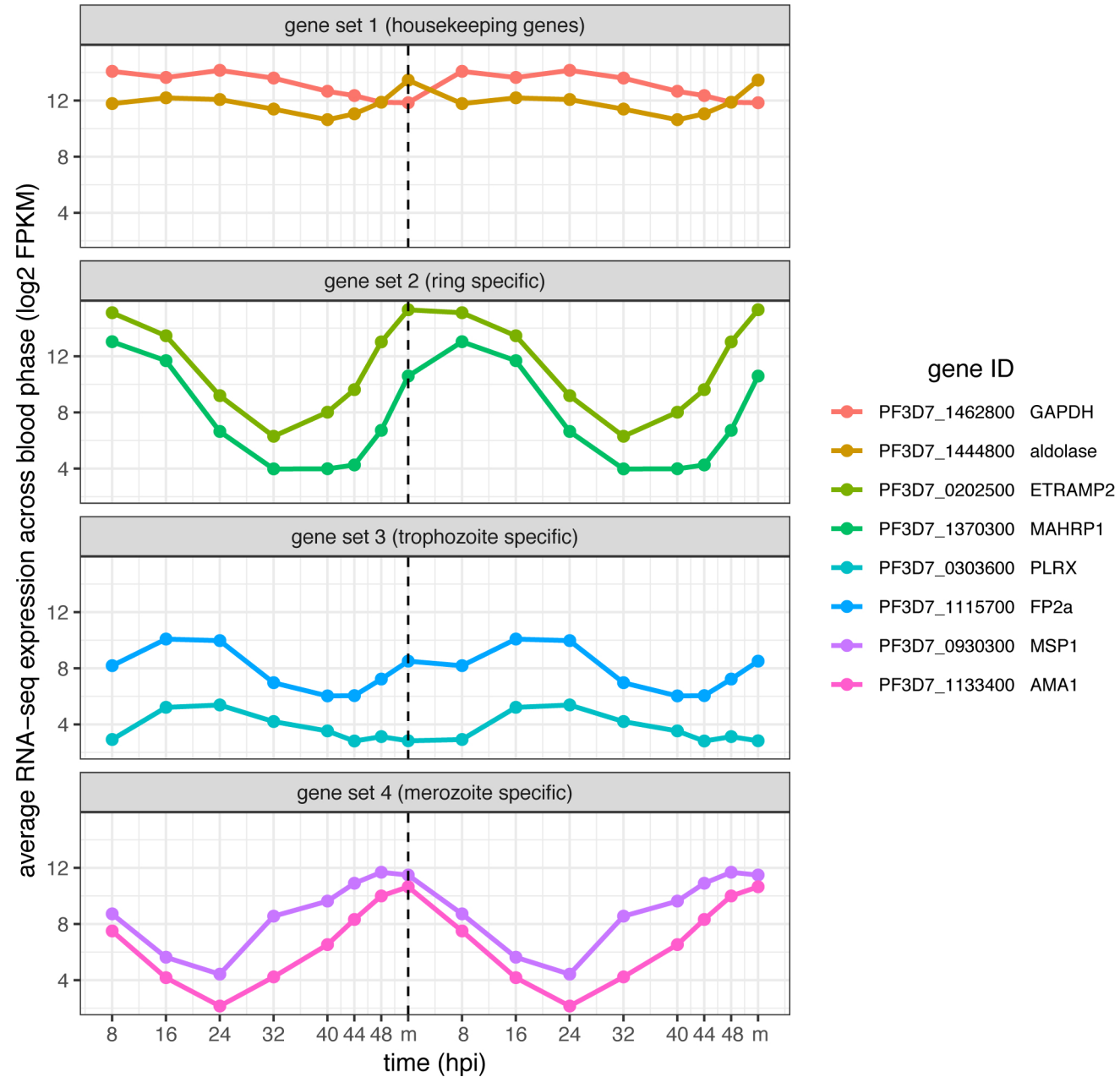

Supplement: FIG S4 [file mBio.01500-19-sf004.pdf]

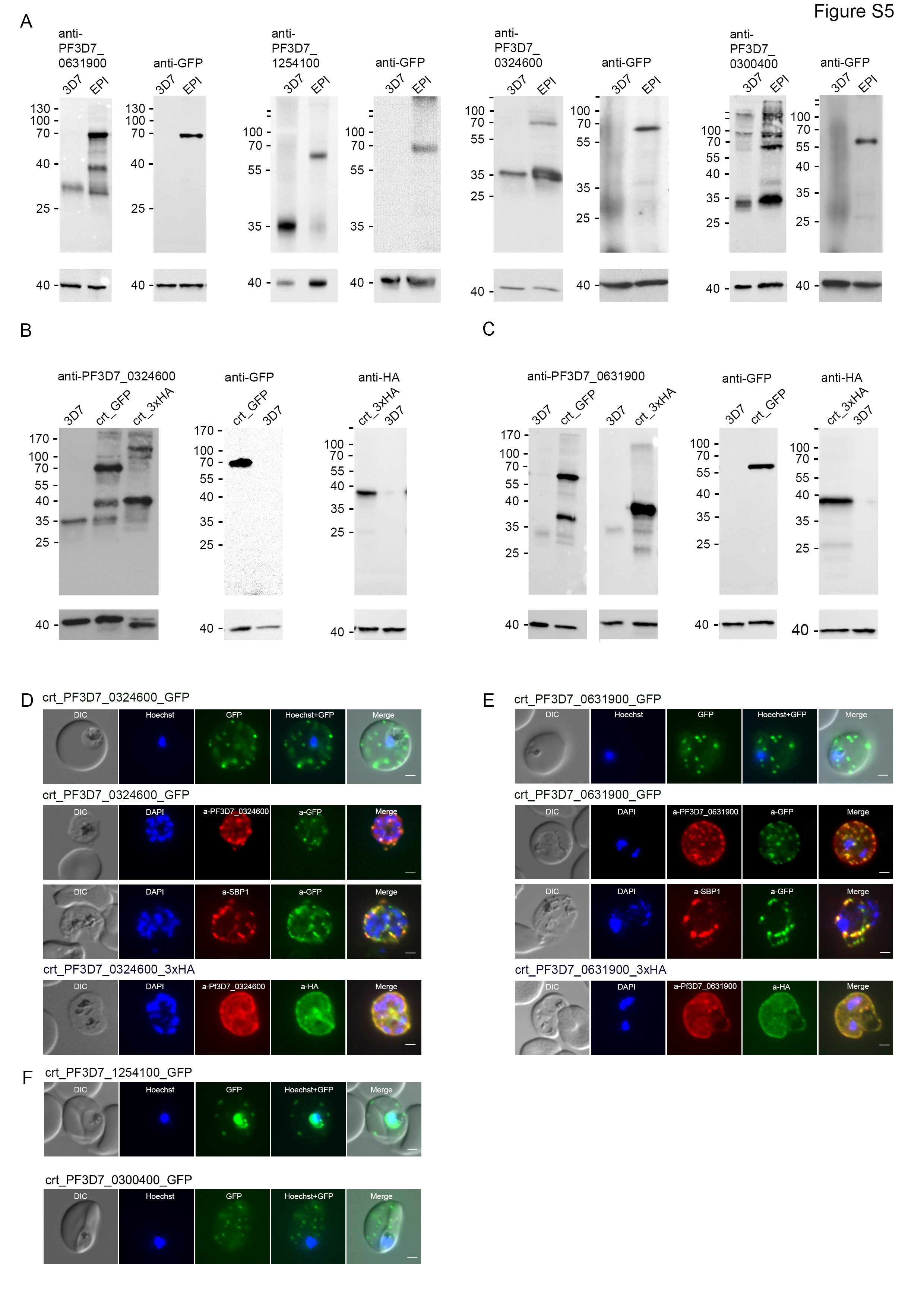

Supplement: FIG S5 [file mBio.01500-19-sf005.tif]

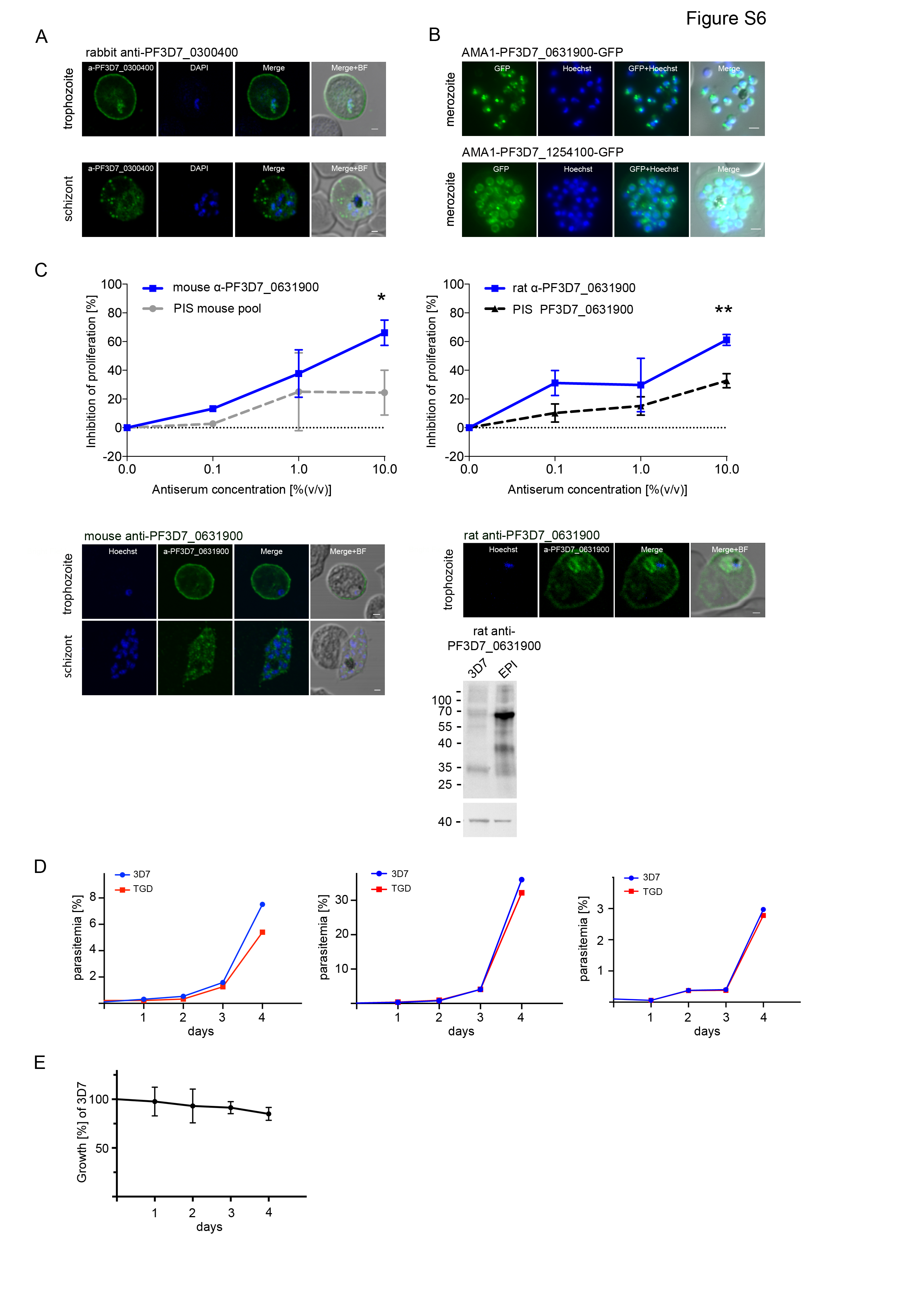

Supplement: FIG S6 [file mBio.01500-19-sf006.tif]
