# Supplementary material for: Dissecting the Gene Expression, Localization, Membrane Topology, and Function of the Plasmodium falciparum STEVOR Protein Family
Source: mBio. 2019 Jul 30;10(4):e01500-19. doi: 10.1128/mBio.01500-19 (PMC6667621; doi:10.1128/mBio.01500-19)
Supplement: TABLE S1 [file mBio.01500-19-st001.pdf]

Table S1: Summary of metadata and experiment design for RNA-seq data sets analyzed in this study

Study (PMID)	Accession	developmental stages / time points of harvest (hpi)	RNA extraction method	RNA selection method	Library layout	Read types	Library strand	Replicates	Platform
this study	E-MTAB-7731	8, 16, 24, 32, 40, 44, 48, m	Column chromatography (PureLink RNA Mini Kit) in conjunction with Guanidinium thiocyanate (Trizol)	oligo(dT) beads and random priming	paired	2 x 100 bp	strand-specific	yes (biological)	Illumina HiSeq 4000
Otto et *al*. 2010	PRJEB2015	0, 8, 16, 24, 32, 40, 48	Guanidinium thiocyanate (Trizol)	streptavidin beads, oligo(dT) and random priming	paired	2 x 54 bp<br>2 x 37 bp	not-stranded	no	Illumina Genome Analyzer<br>Illumina Genome Analyzer II
Bartfai et *al*. 2010	PRJNA133177	5, 10, 15, 20, 25, 30, 35, 40	column chromatography (RNeasy Mini Kit, Qiagen)	poly(A) selection	single	1 x 75 bp	not-stranded	no	Illumina Genome Analyzer II
Lopez-Barragan et *al*. 2011	PRJNA76935	8, 19, 30, 42	Guanidinium thiocyanate (Trizol)	oligo(dT) and random priming	single<br>paired	1 x 36 bp<br>2 x 45 bp	not-stranded<br>strand-specific	no	Illumina Genome Analyzer<br>Illumina Genome Analyzer II
Sorber et *al*. 2011	PRJNA79781	11, 22, 33, 44	Guanidinium thiocyanate (Trizol)	poly(A) selection	single<br>paired	1 x 42 bp<br>2 x 42 bp	strand-specific	yes (technical)	Illumina Genome Analyzer II
Bunnik et *al*. 2013	PRJNA198874	0, 18, 36	Guanidinium thiocyanate (Trizol)	oligo(dT) and random priming	paired	2 x 50 bp	not-stranded	no	Illumina HiSeq 2000
Siegel et *al*. 2014	PRJEB3309	10, 20, 30, 40	column chromatography (miRNeasy Mini Kit, Qiagen)	poly(A) selection	single	1 x 60 bp	strand-specific	no	Illumina HiSeq 2000
Broadbent et *al*. 2015	PRJNA246463	6, 8, 14, 20*, 28*, 32*, 36, 40*, 44, 48	column chromatography (RNeasy Midi and Mini Kit, Qiagen) in conjunction with Guanidinium thiocyanate (Trizol)	rRNA depletion	paired	2 x 100 bp	strand-specific	yes (biological)	Illumina HiSeq 2000

*Two biological replicate samples
