# Supplementary material for: Dissecting the Gene Expression, Localization, Membrane Topology, and Function of the Plasmodium falciparum STEVOR Protein Family
Source: mBio. 2019 Jul 30;10(4):e01500-19. doi: 10.1128/mBio.01500-19 (PMC6667621; doi:10.1128/mBio.01500-19)
Supplement: TABLE S2 [file mBio.01500-19-st002.pdf]

Table S2: Summary of RNA-seq mapping statistics for Plasmodium falciparum 3D7 RNA-seq data set generated in this study.

Sample	Replicate	Sample ID [ENA run accession]	Total reads	Uniquely mapped reads	% uniquely mapped reads	Multi-mapping reads	% multi-mapping reads	Unmapped reads	% unmapped reads
Bio1_08_hpi	r1	ERS3200747	13718428	13087788	95,40	327494	2,39	303146	2,21
Bio1_16_hpi	r1	ERS3200748	11722979	11117765	94,84	344628	2,94	260586	2,23
Bio1_24_hpi	r1	ERS3200749	11836647	11258816	95,12	344386	2,91	233445	1,98
Bio1_32_hpi	r1	ERS3200750	7854343	7504316	95,54	189913	2,42	160114	2,04
Bio1_40_hpi	r1	ERS3200751	9618425	9142711	95,05	220856	2,30	254858	2,65
Bio1_44_hpi	r1	ERS3200752	13522967	12733103	94,16	345797	2,56	444067	3,28
Bio1_48_hpi	r1	ERS3200753	11791690	11196201	94,95	273462	2,32	322027	2,73
Bio1_merozoites	r1	ERS3200754	10598019	10070503	95,02	306180	2,89	221336	2,09
Bio2_08_hpi	r2	ERS3200755	11220995	10650972	94,92	311643	2,78	258380	2,31
Bio2_16_hpi	r2	ERS3200756	15011903	14232874	94,81	425091	2,83	353938	2,36
Bio2_24_hpi	r2	ERS3200757	9370631	8939800	95,40	238819	2,55	192012	2,05
Bio2_32_hpi	r2	ERS3200758	11292773	10808104	95,71	253983	2,25	230686	2,04
Bio2_40_hpi	r2	ERS3200759	10083191	9582466	95,03	259461	2,57	241264	2,39
Bio2_44_hpi	r2	ERS3200760	12362544	11428362	92,44	622574	5,03	311608	2,52
Bio2_48_hpi	r2	ERS3200761	9946718	9413749	94,64	248931	2,50	284038	2,86
Bio2_merozoites	r2	ERS3200762	10054790	9479595	94,28	295002	2,93	280193	2,79
Bio3_08_hpi	r3	ERS3200763	15024078	14262148	94,93	401919	2,67	360011	2,40
Bio3_16_hpi	r3	ERS3200764	14788804	13978757	94,52	446318	3,02	363729	2,46
Bio3_24_hpi	r3	ERS3200765	10372699	9899574	95,44	260207	2,51	212918	2,05
Bio3_32_hpi	r3	ERS3200766	13596681	12944076	95,20	317589	2,34	335016	2,46
Bio3_40_hpi	r3	ERS3200767	11228565	10628669	94,66	297710	2,65	302186	2,69
Bio3_44_hpi	r3	ERS3200768	10840292	10145273	93,59	307927	2,84	387092	3,57
Bio3_48_hpi	r3	ERS3200769	13347167	12516929	93,78	428718	3,21	401520	3,01
Bio3_merozoites	r3	ERS3200770	12308073	11597747	94,23	404328	3,28	305998	2,49
