# Supplementary material for: Dissecting the Gene Expression, Localization, Membrane Topology, and Function of the Plasmodium falciparum STEVOR Protein Family
Source: mBio. 2019 Jul 30;10(4):e01500-19. doi: 10.1128/mBio.01500-19 (PMC6667621; doi:10.1128/mBio.01500-19)
Supplement: FIG S3 [file mBio.01500-19-sf003.pdf]

Figure S3

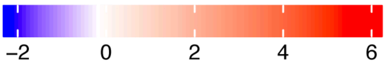

	8 hpi	16 hpi	24 hpi	32 hpi	40 hpi	44 hpi	48 hpi	merozoites
PF3D7_0901600	−0.3	−0.5	−0.1	−0.2	0.0	0.9	1.5	1.5
PF3D7_0500600*	−1.4	−1.4	−0.9	0.2	0.3	1.4	1.5	1.1
PF3D7_0201300	−0.1	0.1	0.5	−0.3	−0.2	−0.1	−0.1	0.6
PF3D7_0101800	0.3	0.9	0.2	−0.3	−0.0	−0.3	−0.3	0.7
PF3D7_0400800	−0.3	0.6	0.5	−0.1	−0.1	−0.1	0.0	0.7
PF3D7_0222800	−0.3	0.2	0.4	−0.3	−0.3	0.2	0.0	−0.3
PF3D7_0732000	−1.0	−0.2	1.8	0.2	−0.2	0.1	−0.7	0.5
PF3D7_0200900*	0.0	0.0	0.0	0.2	0.2	0.2	0.0	0.3
PF3D7_0221400	−1.6	1.2	0.6	−0.4	−0.2	−0.0	0.1	0.0
PF3D7_0900900	−0.2	0.1	0.5	−0.2	−0.2	0.1	0.1	0.4
PF3D7_0401500*	−0.4	1.1	1.8	−0.5	−0.1	−0.3	0.3	0.5
PF3D7_0617600	−1.4	2.6	2.1	0.3	0.4	−0.1	−0.2	−0.8
PF3D7_0200400	0.0	0.3	0.6	0.0	0.2	0.0	0.0	0.0
PF3D7_1254300	0.0	1.3	1.1	−0.4	0.0	−0.2	−0.1	−0.4
PF3D7_1479500	−0.8	2.4	2.3	0.1	−0.1	−0.1	0.1	0.1
PF3D7_1000800*	−1.6	5.0	4.4	0.4	−0.9	−0.2	−0.9	−0.3
PF3D7_1040200	−0.5	4.7	4.2	0.6	0.0	0.1	−0.1	−0.7
PF3D7_0631900	−1.4	3.0	3.5	0.1	−0.4	−0.3	−0.1	0.2
PF3D7_0324600	−1.8	4.0	3.2	−0.2	0.2	0.0	0.2	−1.0
PF3D7_1254100	−0.6	2.0	2.5	−0.7	−0.3	0.3	0.1	−0.1
PF3D7_1479900	−0.3	0.7	1.9	0.0	−0.1	−0.1	−0.3	−0.0
PF3D7_0700400	−0.2	0.7	0.7	0.1	−0.0	−0.2	−0.2	0.0
PF3D7_0300400	−0.4	0.1	0.3	−0.2	0.1	−0.4	−0.1	−0.1
PF3D7_1149900	−1.4	3.8	4.3	0.4	−0.7	0.1	−0.2	0.1
PF3D7_1400700	−1.1	2.0	2.9	0.6	−0.7	−0.2	0.0	0.4
PF3D7_0402600	−0.9	1.3	2.5	0.0	−0.1	−0.2	−0.4	0.1
PF3D7_1100700**	−0.4	1.0	1.6	0.1	−0.2	−0.1	−0.1	0.2
PF3D7_0300900*	−0.4	3.2	1.8	0.4	−0.4	−0.5	−0.3	−0.4
PF3D7_0425500	−0.3	3.5	2.0	−0.7	−0.0	−0.2	0.2	0.7
PF3D7_1254600	0.0	2.0	1.2	0.0	0.0	0.0	0.3	0.3
PF3D7_0700700**	−0.2	1.2	0.9	−0.4	−0.3	−0.3	0.0	0.2
PF3D7_0102100*	1.1	0.1	−0.4	−0.2	−0.4	−0.4	0.7	5.3
PF3D7_0532800*	4.1	3.6	0.8	−0.3	−1.4	−1.6	−0.7	0.3
