# Supplementary material for: Dissecting the Gene Expression, Localization, Membrane Topology, and Function of the Plasmodium falciparum STEVOR Protein Family
Source: mBio. 2019 Jul 30;10(4):e01500-19. doi: 10.1128/mBio.01500-19 (PMC6667621; doi:10.1128/mBio.01500-19)
Supplement: TABLE S3 [file mBio.01500-19-st003.pdf]

Table S4: Oligonucleotides used

New ID	Target or Primer name	Sequence	Purpose
PF3D7_1254100	PFL2610w	For: 5'-GGGAAGCTTGAGAATTATCTAAATAACCATTATA<br>Rev: 5'-GGGGGATCCTTAAGAAGTTGCAGCAGGTACTGTTAA	Cloning for antibody generation
PF3D7_0300400	PFC0025c	For: 5'-GGGAAGCTTGTAAGTTTCATTCAAAACAACACCA<br>Rev: 5'-GGGGGATCCTTAACTAGCAGAAGTTGCACCTGAT	Cloning for antibody generation
PF3D7_0631900	PFF1550w	For: 5'-GGGAAGCTTACCCAAATCCATAATCCAC<br>Rev: 5'-GGGGGATCCTTAAGGGGGAAATGCACCTATAGC	Cloning for antibody generation
PF3D7_0324600	PFC1105w	For: 5'-GGGAAGCTTACAAAAAATCATAATCCGC<br>Rev: 5'-GGGGGATCCTTAACAAGGTTGAAATGTTCCC	Cloning for antibody generation
PF3D7_0631900	PFF1550w	For: 5'-CCGTTGATGCCATCCTTCCT<br>Rev: 5'-CACACGCTTCGGTTGCTTTA	qPCR
PF3D7_1254100	PFL2610w	For: 5'-GTGCGTGCAAATCCTCTATCAC<br>Rev: 5'-ATTGCACCAGCAGAAGTTGC	qPCR
PF3D7_0300400	PFC0025c	For: 5'-CTATTTCTACCGCGGGTGCT<br>Rev: 5'-GCGTAATCAGCAGCACAACC	qPCR
PF3D7_0324600	PFC1105w	For: 5'-TTGCTGCCGTCCTTTCTACC<br>Rev: 5'-TGGTTGCACAAAGAACTGACTG	qPCR
PF3D7_0400800	PFD0035c	For: 5'-TAATGCGTCCACATCCCCTG<br>Rev: 5'-GCAATACCCCAGGGCTGTAA	qPCR
PF3D7_1040200	PF10_0395	For: 5'-AAAACTGCTGCCAAAGGTGC<br>Rev: 5'-TGCCTGCTTGAAGAGCTGAA	qPCR
PF3D7_0617600	PFF0850c	For: 5'-GTAGGTGAACATGGTACAGAACCT<br>Rev: 5'-TGTCATCATCATTTGGGGGCA	qPCR
PF3D7_0221400	PFB0955w	For: 5'-AAGGGTTGCTTTGGAGGTGT<br>Rev: 5'-TCTTTAGCAGCAGTCACAGCA	qPCR
PF3D7_1444800	PF14_0425[1]	For: 5'-TGTACCACCAGCCTTACCAG<br>Rev: 5'-TTCCTTGCCATGTGTTCAAT	qPCR
PF3D7_1300900	MAL13P1.7	For: 5'-TGCAGAACCTATGTCAACGCT<br>Rev: 5'-TGATTTGTCGTATTGATTGGTGTT	qPCR
PF3D7_0115400	PFA0750w	For: 5'-AGGCTGCCTTTGATACCTTGA<br>Rev: 5'-CCGCCTGCAAACATAGATGG	qPCR
PF3D7_0832000	MAL7P1.227	For: 5'-AGTGTACGAAGCTCTGGCTG<br>Rev: 5'-TCTGGGGCTGAAAAAGCACT	qPCR
PF3D7_0832900	MAL7P1.218	For: 5'-CTGCCGTTGGTCCCTTCAAT<br>Rev: 5'-CTGCCTTAACAGCTTCCCCT	qPCR
PF3D7_1372500	MAL13P1.490	For: 5'-GCTGGTAATGTTGCTGCTGG<br>Rev: 5'-ACCAGGGCTTGGGTCAATTT	qPCR
PF3D7_1372800	MAL13P1.505	For: 5'-GCTATTCAAGCAGGTGGTGG<br>Rev: 5'-AAGCTGCAATACCACAGGGG	qPCR
PF3D7_0832400	MAL7P1.223	For: 5'-AAGTGAGATGTTCCCGTGGT<br>Rev: 5'-GACAGTAAAGTGCGCAAGGG	qPCR
PF3D7_0832600	MAL7P1.310	For: 5'-TTGTCCCAAACCCTTGTCGG<br>Rev: 5'-TCAGTAGCAGCCTTCGTAGC	qPCR
PF3D7_0114600	PFA0705c	For: 5'-AGCTTCATACCAAATAACACACAAA<br>Rev: 5'-TGGGTCGTTGCGTTGTGATA	qPCR
PF3D7_0102100	PFA0105w	For: 5'-CCTAAAACGAAGATGTATAGGTGGA<br>Rev: 5'-TGCAGAGTATTTTGCCTTTGCC	qPCR
PF3D7_0101800	PFA0090c	For: 5'-GCGGTTGATGCCCTTGCTA<br>Rev: 5'-GCAGCACAAACTGCACTACC	qPCR
PF3D7_0732000	PF07_0130	For: 5'-GTGCCTAAGAATTGCATCACCT<br>Rev: 5'-ACACAAACTGCTGTACCTACTGA	qPCR
PF3D7_0700400	MAL8P1.217	For: 5'-GGTTCATTTCCAGATGCTGCT<br>Rev: 5'-ACCACCTGATGCACTAACTGA	qPCR
PF3D7_1254600	PFL2635w	For: 5'-AGTGCGTGTGCATCCTTTGT<br>Rev: 5'-TTCCGCCTAAATCACCAGCA	qPCR
PF3D7_1254300	PFL2620w	For: 5'-GATGCAAAACCTATGTCAGCGA<br>Rev: 5'-TGGACTCATGCCCAACTTCA	qPCR
PF3D7_0901600	PFI0080w	For: 5'-AGGCTGCACTTGCTTACTTT<br>Rev: 5'-TGTTGCACCTCCTGCTTGAA	qPCR
PF3D7_0900900	PFI0045c	For: 5'-AAAGGGAGATGTACCCGTGG<br>Rev: 5'-TGAGAGCAGCTTTAGTAGCACA	qPCR
PF3D7_0425500	PFD1220c	For: 5'-TCAACCGACAAATTATCTTCAACAA<br>Rev: 5'-AGACGAAAGGGCACACACAA	qPCR
PF3D7_0402600	PFD0125c	For: 5'-CTGGTTGTGTTGCATGTGTAGG<br>Rev: 5'-GCACTAGAAGTAGTCTTAATTGCCT	qPCR
PF3D7_0200900	PFB0050c	For: 5'-TATAGTGCGTGTGCAGCCTT<br>Rev: 5'-CGCCTAAATCAGCCACCAGA	qPCR
PF3D7_1400700	PF14_0007	For: 5'-AAAAGCCTCTTACAAAAGAACACA<br>Rev: 5'-TGCGGATTATGGTTTTGGGTTT	qPCR
PF3D7_1479500	PF14_0767	For: 5'-GCTGTCATTGGTTCATTTGGAGA<br>Rev: 5'-AGAGCCTGCTGCATTAACTGA	qPCR
PF3D7_0201300	PFB0065w	For: 5'-CATGTAGGTGGACATGTTTCTGA<br>Rev: 5'-GGTACCTACCCGATTTTAACATAAC	qPCR
PF3D7_1149900	PF11_0516	For: 5'-AAAATGGAGATGTACCCGTGCT<br>Rev: 5'-GCAACGTCAGTAGCTCTTGC	qPCR
PF3D7_0200400	PFB0025c	For: 5'-GAAAGGGAGATGTACCCGTG<br>Rev: 5'-TGAAGCAACAGCACTTAGGG	qPCR
PF3D7_1479900	PF14_0771	For: 5'-AGTACATGTGTTGAGGGTGCT<br>Rev: 5'-ACCAAAGCTGCAATACCCCA	qPCR
PF3D7_0222800	PFB1020w	For: 5'-GCAGCAGGAGGTGTAGAATGT<br>Rev: 5'-AAGTAGCACCAAAGCTGCAA	qPCR
PF3D7_1100700	PF11_0013	For: 5'-CGTTGTGTTGGAGGTGTAGG<br>Rev: 5'-AGCCATAAACCAACAGGAGTGT	qPCR
PF3D7_1000800	PF10_0009	For: 5'-TGTGCCTTTTCTTCTGCACT<br>Rev: 5'-GAGCAAGTACTGGGAGCGAG	qPCR
PF3D7_0500600	PFE0030c	For: 5'-TGAGAATGCTGCTGTTCCTATTTT<br>Rev: 5'-AATACCAAAGCTGCAATACCATAAG	qPCR
PF3D7_0401500	PFD0065w	For: 5'-TGACCCAGAACTTAAGGAAATGA<br>Rev: 5'-ACCACCTGTTAATTTTGTTTCGCT	qPCR
PF3D7_0700700	MAL8P1.214	For: 5'-ACTCCTGTTGGTTTATGGTCTCC<br>Rev: 5'-CACAGCAATTGCAATATCATAAGGT	qPCR
PF3D7_0532800	PFE1627c	For: 5'-GTTTGTGCAGAAGTTCTTGGC<br>Rev: 5'-GCAGTAGCAGTAGAAGAAGCAC	qPCR
PF3D7_1133400	PF11_0344	For: 5'-TGGGTAATCCATGGACGGAA<br>Rev: 5'-TGAGTTCCAGCTACTTCAGCA	qPCR
PF3D7_0501300	PFE0065w	For: 5'-TTAGCCGACGAACCAACACA<br>Rev: 5'-TTCGGTTGTCTCTGGTACTGCA	qPCR
PF3D7_1218600	PFL0900c	For: 5'-TTCAAAACACGAAGTGGAACAAC<br>Rev: 5'-AATTCTCTGCAGCAAGTCGC	qPCR
PF3D7_0631900	PFF1550w	For: 5'-GCGGCCGCTAAATGTTATTGTTTAACTTTTTAATAAATGTTTTAGTA<br>Rev: 5'-ACGCGTAGGTTCTGCATCATTACCACCTTTATA	Cloning for TGD
PF3D7_0631900	PFF1550w	For: 5'-TGAAGATGTATTACCTTAAA<br>Rev: 5'-TGTAGTTAATGAACCACCAT	Integration check PCR TGD
	pARL sense 55	For: 5'-GGAATTGTGAGCGGATAACAATTTCACACAGG	Integration check PCR TGD/Sequencing
	NEO 40 rv[2]	Rev: 5'-CGAATAGCCTCTCCACCCAAG	Sequencing
	gfp tag rv	Rev: 5'-TTTTGTTGATAATGGTCTGC	Integration check PCR/Sequencing
	crt sense	For: 5'-CCGTTAATAATAAATACACGCAGTC	Sequencing
	all stevors[3]	For: 5'-TCCRCATTATCAYAATGAYCCAG	Sequencing
	ama1 sense	For: 5'-CCTAATAATTTATTTGATAATTTTTC	Sequencing
PF3D7_0324600	PFC1105w	For: 5'-GGGGCGGCCGCGCACAAAGAACAACGATAAATTC<br>Rev: 5'-GGGTACCCTTGCATAAATGTTTCTTGCATTCAT	Cloning for overexpression
PF3D7_1254100	PFL2610w	For: 5'-GGGGCGGCCGCGACCAAAAGAGCAACGATAAATTC<br>Rev: 5'-GGTACCCTTACATAAATGTTTCTTGCATTCATG	Cloning for overexpression
PF3D7_0631900	PFF1550w	For: 5'-GGGGGTACCATGAAGATGTATWACCTTAAAATGTTATTG<br>Rev: 5'-GGGCCTAGGCTTACATAAATGTTTCTTGCATTSATG	Cloning for overexpression
PF3D7_0300400	PFC0025c	For: 5'-GGGGGTACCATGAAGATGTATTATCTWAAAATGTTATTG<br>Rev: 5'-GGGCCTAGGCTTACATAAATGTTTCTTGCATTSATG	Cloning for overexpression
PF3D7_0631900	QC35_PFF1550w_glyc_loop2_for<br>QC35_PFF1550w_glyc_loop2_rev	For: 5'-CTCCAGTTCATTATTTCATATATTTCATGGTGGTTCAAACACTACAGCTCTTAAAGCAAC<br>Rev: 5'-GTTGCTTTAAGAGCTGTAGTGTTTGAACCACCATGAAATATATGAAATAATGAACTGGAG	*in vitro* translation assays
PF3D7_0631900	PFF1550w_Sma_rev	Rev: 5'-TCTCTCCCCGGGCTTACATAAATGTTTCTTGCATTCATG	*in vitro* translation assays
PF3D7_0631900	QC39PFF1550wStoprev	Rev: 5'-CGAACCTGACGGGATCTGTTACCCGGGCTTACATAAATG	*in vitro* translation assays
PF3D7_0631900	NcoSPKpnmatPFF1550wfor	For: 5'-GAGAGACCATGgctAAGTTGCTGCTGCTCCTCTTGTTGCTCCTGTTACCATCTGCACAGGCAGCTGATCAAAGTGGTAC<br>CGCACAAACCCAAATCCATAATCCAC	*in vitro* translation assays
PF3D7_0631900	PFF1550wdelGlyc2for<br>PFF1550wdelGlyc2rev	For: 5'-GATCCATATGAACAATTACAAGAAGTAGTAGAAAAAAGTGGAACAAAATATAAAGGTG<br>Rev: 5'-CACCTTTATATTTTGTTCCACTTTTTTCTACTACTTCTTGTAATTGTTCATATGGATC	*in vitro* translation assays
PF3D7_0631900	PFF1550wdelGlyc3for<br>PFF1550wdelGlyc3rev	For: 5'-CAAACATATGAAGAAGTGTTTGGTAGCGAAAGTGATATGTTGAAGTC<br>Rev: 5'-GACTTCAACATATCACTTTCGCTACCAAACACTTCTTCATATGTTTG	*in vitro* translation assays

[1] Salanti et al.. 2003
[2] Birnbaum et al., 2017
[3] Bachmann et al.. 2012
