# Supplementary material for: Dissecting the Gene Expression, Localization, Membrane Topology, and Function of the Plasmodium falciparum STEVOR Protein Family
Source: mBio. 2019 Jul 30;10(4):e01500-19. doi: 10.1128/mBio.01500-19 (PMC6667621; doi:10.1128/mBio.01500-19)
Supplement: TABLE S4 [file mBio.01500-19-st004.pdf]

Table S4A: *stevor* ALDEx2 effect sizes

old gene ID	new gene ID	description	08_hpi	16_hpi	24_hpi	32_hpi	40_hpi	44_hpi	48_hpi	merozoites	kw.eBH	glm.eBH	kw.signif_code	glm.signif_code
PFA0105w	PF3D7_0102100	stevor, pseudogen	0,97179281	-0,85793639	-0,85876509	0,11539355	-0,34429938	0,18963723	1,35175776	1,71948515	0,02445597	0,00155191	*	** P<0.01
PFA0090c	PF3D7_0101800	stevor	0,20551015	0,29911922	0,11574062	-0,88430663	0,36144602	-0,3927862	0,14572157	0,79382918	0,27526776	0,1771561		
PF07_0130	PF3D7_0732000	stevor	1,51513743	0,91117444	1,0837584	-1,6584449	-0,57090025	0,62046571	-0,7786807	0,94187486	0,06450879	0,01374045		* P<0.05
MAL8P1.217	PF3D7_0700400	stevor	0,5248993	0,86360514	0,34715388	-1,07781973	-0,20967352	-0,01762897	-0,29071524	0,87225674	0,17485816	0,09888414		
PFL2635w	PF3D7_1254600	stevor	0,23161995	0,80629703	0,10828259	-1,37083342	-0,18545756	0	0,68260852	0,22340577	0,12449836	0,06024083		
PFL2620w	PF3D7_1254300	stevor	-0,37967372	1,1956399	0,5355127	-1,48029325	0,30315915	-0,12352442	0,0091059	0,06938969	0,08453317	0,0356333		* P<0.05
PFL2610w	PF3D7_1254100	stevor	0,25436244	0,78397346	0,52846548	-1,52902908	0,40432287	0,52509187	-0,19463745	-0,1693599	0,1887106	0,08483222		
PFI0080w	PF3D7_0901600	stevor	0,66269636	-0,03832755	0,56425183	0,09785183	0,46220278	0,66474776	0,90146447	-0,00978154	0,09202804	0,07313226		
PFI0045c	PF3D7_0900900	stevor	0,39000004	0,66219828	0,33029246	-1,23470692	0,16343958	0,42564973	0,05854207	0,20648571	0,24944552	0,15064281		
PFF1550w	PF3D7_0631900	stevor	0,66583872	1,41366825	0,34461705	-4,04421261	-0,17552351	0,01223322	0,29285293	-0,06135791	0,10876021	0,00427301		** P<0.01
PFF0850c	PF3D7_0617600	stevor	0,33339321	1,3042884	0,34955713	-1,19553292	-0,1481772	-0,49571533	0,05382683	-0,50769787	0,05341221	0,00696916		** P<0.01
PFD1220c	PF3D7_0425500	stevor	0,78240183	2,17006588	0,02034722	-2,41842492	0,72048578	0,21791558	0,29422719	0,70323359	0,03484565	0,00025747	*	*** P<0.001
PFD0125c	PF3D7_0402600	stevor	1,19268058	1,03888124	0,71714285	-2,0105314	-0,18866211	0,03500686	-0,09933917	0,74816336	0,12102732	0,05072159		
PFD0035c	PF3D7_0400800	stevor	0,6078735	0,71742524	0,11466631	-0,91259761	0,0283024	0,22082261	0,05630277	1,14752397	0,14281978	0,10117466		
PFC0025c	PF3D7_0300400	stevor	0,31401769	0,60790618	0,34245248	-1,01684477	0,04919611	-0,07594626	0,21976067	0,21937864	0,48954743	0,32653745		
PFC1105w	PF3D7_0324600	stevor	0,65469831	1,86060125	0,26705559	-1,87604658	-0,0698664	-0,13996075	0,03274829	-0,65844398	0,03365714	0,00026778	*	*** P<0.001
PFB0050c	PF3D7_0200900	stevor, pseudogen	0,33255726	0,30210921	0,17480463	-0,12383681	0,08079943	-0,09146047	0,03765817	0,43619556	0,68101091	0,54794215		
PF14_0007	PF3D7_1400700	stevor	1,0330706	1,42545603	0,59701263	-1,82144845	-0,35110561	0,38805694	0,37538933	0,38802156	0,08328846	0,00407913		** P<0.01
PF14_0767	PF3D7_1479500	stevor	1,01068629	1,74129308	0,10801206	-2,24103767	-0,10308032	0,09254941	0,2143185	0,14580215	0,06255865	0,00486616		** P<0.01
PFB0065w	PF3D7_0201300	stevor	0,84469325	0,47407495	0,43980481	-1,6305234	0,36413692	-0,14035762	0,11815415	1,258795	0,1204861	0,07399617		
PF10_0395	PF3D7_1040200	stevor	-0,07520313	1,38436099	0,04325597	-3,81462265	-0,3542098	0,03074225	-0,22803199	-0,45151538	0,05437007	0,00081023		*** P<0.001
PF11_0516	PF3D7_1149900	stevor	1,031607	1,81685581	0,54011107	-2,56571141	-1,19140516	0,99363487	-0,58686362	0,5550704	0,02794016	9,8638E-05	*	*** P<0.001
PFB0025c	PF3D7_0200400	stevor	0,17095893	0,48714506	0,68841697	-0,96499321	0,28800272	-0,14592406	-0,22628114	0,41035222	0,26033231	0,19731164		
PF14_0771	PF3D7_1479900	stevor	0,11285207	0,68407245	0,86460109	-1,66758708	-0,12652028	0,26747143	-0,3135836	0,32986484	0,15009867	0,07532452		
PFB1020w	PF3D7_0222800	stevor	-0,07946805	0,55014892	0,4686487	-1,12709049	-0,06885198	0,90436017	-0,33356675	-0,26964073	0,21320351	0,11951369		
PF11_0013	PF3D7_1100700	stevor-like	0,59818929	0,9699397	0,49921525	-1,81859087	-0,39704518	0,00655241	0,04959779	0,39332141	0,11617681	0,07904628		
PF10_0009	PF3D7_1000800	stevor, pseudogen	0,25404308	1,50693439	-0,09305685	-3,19546927	-0,50175194	0,27500745	-0,21686328	0,40643757	0,08576225	6,14E-05		*** P<0.001
PFB0955w	PF3D7_0221400	stevor	1,26446149	1,61367739	0,15651854	-0,91248252	0,17935222	0,28583662	0,26858819	0,11053714	0,13109518	0,01260348		* P<0.05
PFE0030c	PF3D7_0500600	stevor, pseudogen	0,99470656	-0,09467563	0,67086832	1,27428471	0,72067939	0,45280164	0,34674687	-0,61532621	0,0283543	0,00417709	*	** P<0.01
PFD0065w	PF3D7_0401500	stevor, pseudogen	0,69606367	1,0276888	0,72804375	-2,32104201	0,57014992	0,04248974	0,62436983	0,08577432	0,11320497	0,01795626		* P<0.05
MAL8P1.214	PF3D7_0700700	stevor-like	0,04662583	1,28811839	0,09391743	-1,54140486	0,01011688	-0,2797656	0,93615277	0,02954438	0,06676147	0,00865412		** P<0.01
PFC0045c	PF3D7_0300900	stevor-like	-0,24987992	1,64144428	-0,33183848	-1,90824832	-0,03523176	-0,15642365	0,37451533	-0,23744059	0,07723949	0,00631741		** P<0.01
PFE1627c	PF3D7_0532800	stevor, pseudogen	-2,46640733	-0,10433756	-3,37395551	-1,61604851	-0,87802515	-0,39218917	1,32776472	1,46768019	0,02170407	1,7356E-08	*	*** P<0.001

Table S4B: *stevor* ALDEx2 overlap

old gene ID	new gene ID	description	08_hpi	16_hpi	24_hpi	32_hpi	40_hpi	44_hpi	48_hpi	merozoites	kw.eBH	glm.eBH	kw.signif_code	glm.signif_code
PFA0105w	PF3D7_0102100	stevor, pseudogen	0,21875093	0,19689226	0,14062668	0,43750014	0,30051867	0,39378263	0,04167315	0,01564272	0,02445597	0,00155191	*	** P<0.01
PFA0090c	PF3D7_0101800	stevor	0,36979198	0,30208386	0,46113998	0,15625147	0,30569999	0,26041737	0,44041464	0,16666802	0,27526776	0,1771561		
PF07_0130	PF3D7_0732000	stevor	0,03125874	0,17098575	0,08333642	0,0003654	0,21243619	0,17708459	0,16062317	0,12953552	0,06450879	0,01374045		* P<0.05
MAL8P1.217	PF3D7_0700400	stevor	0,25520906	0,14583494	0,32642531	0,10416906	0,3886013	0,492228	0,34895871	0,13541843	0,17485816	0,09888414		
PFL2635w	PF3D7_1254600	stevor	0,39378263	0,17708459	0,45833343	0,02073856	0,38341996	0	0,19270945	0,39378263	0,12449836	0,06024083		
PFL2620w	PF3D7_1254300	stevor	0,30729218	0,13541843	0,29166724	0,00526045	0,34895871	0,4270835	0,49740933	0,46113998	0,08453317	0,0356333		* P<0.05
PFL2610w	PF3D7_1254100	stevor	0,3730573	0,22798014	0,22279882	0,0777235	0,35233197	0,30569999	0,42487064	0,4196893	0,1887106	0,08483222		
PFI0080w	PF3D7_0901600	stevor	0,20312604	0,47150265	0,21354263	0,46354175	0,28497468	0,25906806	0,13541843	0,48704666	0,09202804	0,07313226		
PFI0045c	PF3D7_0900900	stevor	0,29015601	0,22798014	0,31770881	0,0572963	0,4041453	0,28497468	0,47916671	0,39896397	0,24944552	0,15064281		
PFF1550w	PF3D7_0631900	stevor	0,19270945	0,07812831	0,28125061	0,0003654	0,42487064	0,492228	0,38541694	0,47668399	0,10876021	0,00427301		** P<0.01
PFF0850c	PF3D7_0617600	stevor	0,30569999	0,09326694	0,43005197	0,11458548	0,43005197	0,29166724	0,47916671	0,23834278	0,05341221	0,00696916		** P<0.01
PFD1220c	PF3D7_0425500	stevor	0,1502606	0,0003654	0,49479168	0,0003654	0,17708459	0,4196893	0,3750003	0,23958414	0,03484565	0,00025747	*	*** P<0.001
PFD0125c	PF3D7_0402600	stevor	0,06250422	0,1510432	0,18652965	0,0003654	0,39583358	0,48437504	0,45833343	0,18750116	0,12102732	0,05072159		
PFD0035c	PF3D7_0400800	stevor	0,26041737	0,14062668	0,46113998	0,13989805	0,48186533	0,37823863	0,45595865	0,06250422	0,14281978	0,10117466		
PFC0025c	PF3D7_0300400	stevor	0,36458366	0,21875093	0,32812544	0,13471678	0,46875007	0,45077731	0,42187518	0,3886013	0,48954743	0,32653745		
PFC1105w	PF3D7_0324600	stevor	0,16580446	0,0003654	0,43750014	0,0003654	0,47395839	0,44559598	0,48186533	0,22279882	0,03365714	0,00026778	*	*** P<0.001
PFB0050c	PF3D7_0200900	stevor, pseudogen	0,29533734	0,38541694	0,4270835	0,45312511	0,44791679	0,43750014	0,46113998	0,2760423	0,68101091	0,54794215		
PF14_0007	PF3D7_1400700	stevor	0,11458548	0,05208846	0,22798014	0,0003654	0,31606265	0,38020862	0,32291712	0,3419693	0,08328846	0,00407913		** P<0.01
PF14_0767	PF3D7_1479500	stevor	0,09896087	0,02073856	0,39378263	0,0003654	0,4041453	0,43750014	0,3575133	0,4270835	0,06255865	0,00486616		** P<0.01
PFB0065w	PF3D7_0201300	stevor	0,14062668	0,2760423	0,23437584	0,01044326	0,33678797	0,44041464	0,44270846	0,05208846	0,1204861	0,07399617		
PF10_0395	PF3D7_1040200	stevor	0,44041464	0,04663783	0,45833343	0,0003654	0,33160664	0,48704666	0,3886013	0,22395923	0,05437007	0,00081023		*** P<0.001
PF11_0516	PF3D7_1149900	stevor	0,0777235	0,00526045	0,23834278	0,0003654	0,06218036	0,1502606	0,28645892	0,24870542	0,02794016	9,8638E-05	*	*** P<0.001
PFB0025c	PF3D7_0200400	stevor	0,42187518	0,25388674	0,16666802	0,09844813	0,33160664	0,4041453	0,36269463	0,27979336	0,26033231	0,19731164		
PF14_0771	PF3D7_1479900	stevor	0,45312511	0,19171096	0,0937527	0,0003654	0,4270835	0,36787597	0,33160664	0,34375039	0,15009867	0,07532452		
PFB1020w	PF3D7_0222800	stevor	0,46632132	0,23437584	0,28497468	0,07254241	0,45833343	0,0937527	0,32642531	0,3750003	0,21320351	0,11951369		
PF11_0013	PF3D7_1100700	stevor-like	0,20833433	0,14507932	0,25000075	0,01564272	0,30569999	0,49479168	0,46632132	0,25000075	0,11617681	0,07904628		
PF10_0009	PF3D7_1000800	stevor, pseudogen	0,42487064	0,01044326	0,4270835	0,0003654	0,38341996	0,3886013	0,39896397	0,32642531	0,08576225	6,14E-05		*** P<0.001
PFB0955w	PF3D7_0221400	stevor	0,02605221	0,02073856	0,45833343	0,12953552	0,39896397	0,35416703	0,38020862	0,44559598	0,13109518	0,01260348		* P<0.05
PFE0030c	PF3D7_0500600	stevor, pseudogen	0,08290462	0,45595865	0,09896087	0,10362933	0,23834278	0,33854207	0,24479245	0,12500194	0,0283543	0,00417709	*	** P<0.01
PFD0065w	PF3D7_0401500	stevor, pseudogen	0,21875093	0,21243619	0,23834278	0,0003654	0,26424938	0,47395839	0,22798014	0,46632132	0,11320497	0,01795626		* P<0.05
MAL8P1.214	PF3D7_0700700	stevor-like	0,47668399	0,05208846	0,44559598	0,05699943	0,49479168	0,34375039	0,12953552	0,48704666	0,06676147	0,00865412		** P<0.01
PFC0045c	PF3D7_0300900	stevor-like	0,3575133	0,02084658	0,34895871	0,0003654	0,492228	0,45312511	0,32124398	0,35937535	0,07723949	0,00631741		** P<0.01
PFE1627c	PF3D7_0532800	stevor, pseudogen	0,0003654	0,41145854	0,0003654	0,01038915	0,16666802	0,29687555	0,0572963	0,01564272	0,02170407	1,7356E-08	*	*** P<0.001
